# Fast and accurate relatedness estimation from high-throughput sequencing data in the presence of inbreeding

**DOI:** 10.1093/gigascience/giz034

**Published:** 2019-04-30

**Authors:** Kristian Hanghøj, Ida Moltke, Philip Alstrup Andersen, Andrea Manica, Thorfinn Sand Korneliussen

**Affiliations:** 1 Centre for GeoGenetics, Natural History Museum of Denmark, University of Copenhagen, 1350 Copenhagen K, Denmark; 2 Université de Toulouse, University Paul Sabatier (UPS), Laboratoire AMIS, CNRS UMR 5288, Toulouse, France; 3 Department of Biology, University of Copenhagen, Ole Maaløes Vej 5, 2200 Copenhagen, Denmark; 4 Department of Zoology, University of Cambridge, Downing Street, Cambridge CB2 3EJ, UK

**Keywords:** relatedness estimation, inbreeding, Jacquard coefficients, high-throughput sequencing data, genotype likelihood, next-generation sequencing, threading, population genetics

## Abstract

**Background:**

The estimation of relatedness between pairs of possibly inbred individuals from high-throughput sequencing (HTS) data has previously not been possible for samples where we cannot obtain reliable genotype calls, as in the case of low-coverage data.

**Results:**

We introduce ngsRelateV2, a major revision of ngsRelateV1, a program that originally allowed for estimation of relatedness from HTS data among non-inbred individuals only. The new revised version takes into account the possibility of individuals being inbred by estimating the 9 condensed Jacquard coefficients along with various other relatedness statistics. The program is threaded and scales linearly with the number of cores allocated to the process.

**Conclusion:**

The program is available as an open source C/C++ program under the GPL license and hosted at https://github.com/ANGSD/ngsRelate. To facilitate easy analysis, the program is able to work directly on the most commonly used container formats for raw sequence (BAM/CRAM) and summary data (VCF/BCF).

## Introduction

Being able to estimate how closely related 2 individuals are and whether they are inbred is important in several different fields ranging from conservation genetics to medical genetics. For this purpose, numerous coefficients, such as the kinship coefficient and inbreeding coefficients, have been defined and many programs for estimating these coefficients have been proposed.

Notably, the genetic relationship between 2 individuals can be quantified by the extent to which the 2 individuals share their alleles via identity by descent (IBD), i.e., are identical as a result of recent common ancestry. More specifically, for 2 diploid individuals, and thus 4 alleles, there are 15 distinct possible IBD sharing patterns at any given site (detailed identity states). If we ignore the maternal or paternal origin of the alleles, these 15 detailed states can be collapsed into 9 condensed states [1] (here denoted *j*_1_, *j*_2_,…, *j*_9_), and their corresponding frequencies in the genome of 2 individuals are called the condensed Jacquard coefficients (here denoted $\mathbf {J_1},\mathbf {J_2},...,\mathbf {J_9}$). These condensed coefficients provide a comprehensive description of the common ancestry between 2 individuals that can be used to infer their familial relationship. Furthermore, many other commonly used coefficients, such as the kinship coefficient and inbreeding coefficients, can be expressed as linear combinations of the 9 condensed Jacquard coefficients.

In the specific case in which neither individual is inbred, only 3 of the condensed Jacquard coefficients can be positive, namely, $\mathbf {J_7},\mathbf {J_8}$, and $\mathbf {J_9}$, which are often also denoted $\mathbf {k_2},\mathbf {k_1}$, and $\mathbf {k_0}$, respectively, and known as Cotterman coefficients [2]. Numerous approaches, based on either method of moments (e.g., [3]) or maximum-likelihood estimation (e.g., [4]), have been devised to estimate these 3 quantities assuming that the rest are zero and thus that the individuals are not inbred. This includes commonly used methods such as PLINK and KING [3,5]. Importantly, these methods can lead to wrong estimates and conclusions if applied to inbred individuals because the assumption that only *J*_7_, *J*_8_, and *J*_9_ can be positive is violated. Hence, in the presence of inbreeding one needs to estimate all 9 coefficients. Several methods for doing this have been proposed [6–8]. However, few current tools allow the user to do this and the few that do all require high-quality genotype data as input (e.g., [8,9]). They therefore cannot be applied to high-throughput (HTS) data of low depth, which are sometimes the only data available. Until recently the same was the case for all the methods for estimating relatedness between non-inbred individuals. For example, both PLINK and KING only work for genotype data. However, recently a few methods that can be applied to low-depth sequencing data have been developed [10,11]. One of these is ngsRelate [11] (hereafter referred to as ngsRelateV1), which works by integrating over every possible genotypic configuration and assigning these a probability given by their genotype likelihood. We here extend this software (hereafter referred to as ngsRelateV2) so that it allows the user to infer all 9 Jacquard coefficients and thus allows for inference of relatedness in the presence of inbreeding, as well as inference of the inbreeding coefficients for both individuals.

## Materials and Methods

The underlying statistical framework is similar to that from ngsRelateV1 [11]. Given 2 individuals, *i* and *j*, from the same homogeneous population, we let $D_l^i$ and $D_l^j$ denote the observed HTS data at a biallelic locus *l*, and $G_l^i$ and $G_l^j$ denote the true, unobserved genotypes at the same locus. Furthermore, we let *f*_*l*_ denote the allele frequency at locus *l* in the relevant population and *X*_*l*_ denote the unobserved IBD state of the 2 individuals at locus *l*. Using this notation we can write the likelihood of the condensed Jacquard coefficients, $\mathbf {J}=(J_1,J_2,J_3,J_4,J_5,J_6,J_7,J_8,J_9)$, for *L* independent (i.e., unlinked) biallelic loci as

\begin{equation*}
L(J|D^i,D^j,f^A) = \prod _{l=1}^L\sum _{m\in \mathbf {J}}P(D_l^i,D_l^j\mid X_l=m,f_l^A)P(X_l=m|J).
\end{equation*}

Notably, here *P*(*X*_*l*_ = *m*∣*J*) = *J*_*m*_ and $P(D_l^i,D^j_l\mid X_l=m,f_l^A)$ can be rewritten as follows:

\begin{equation*}
P(D_l^i,D^j_l\mid X_l = m,f_l^A) \\
\quad = \sum _{G_l^i,G_l^j \in \lbrace 0,1,2\rbrace ^2}P(D_l^i\mid G_l^i)P(D_l^j\mid G_l^j)P(G_l^i,G_l^j\mid f_l^A,X_l=m),
\end{equation*}

where $P(D_l^i|G_l^i)$ and $P(D_l^j|G_l^j)$ denote the per individual genotype likelihoods for a biallelic locus *l*, which can be calculated from the sequencing data and $P(G_l^i,G_l^j\mid f_l^A)$ is given from Table 1. We use this likelihood function as a basis for performing maximum-likelihood estimation. A number of useful estimates can be calculated directly from *J*, such as relatedness [*R* = *J*_1_ + *J*_7_ + 0.75(*J*_3_ + *J*_5_) + 0.5_J__8_], defined as the proportion of homologous alleles IDB [12], and per individual inbreeding coefficients, *F*_1_ and *F*_2_ (as in Vieira et al. [13]).

**Table 1:** Probabilities for various allelic states, given modes of IDB from Table 1 in Anderson and Weir [8], with triallelic sites disregarded

Allelic state	*J*_1_	*J*_2_	*J*_3_	*J*_4_	*J*_5_	*J*_6_	*J*_7_	*J*_8_	*J*_9_
*A*_*i*_ *A*_*i*_ *A*_*i*_ *A*_*i*_	*p*_*i*_	$p_i^2$	$p_i^2$	$p_i^3$	$p_i^2$	$p_i^3$	$p_i^2$	$p_i^3$	$p_i^4$
*A*_*i*_ *A*_*i*_ *A*_*j*_ *A*_*j*_	0	*p*_*i*_ *p*_*j*_	0	*p*_*i*_ *p*_*j*_	0	$p_i^2p_j$	0	0	$p_i^2p_j^2$
*A*_*i*_ *A*_*i*_ *A*_*i*_ *A*_*j*_	0	0	*p*_*i*_ *p*_*j*_	$2p_i^2p_j$	0	0	0	$p_i^2p_j$	$2p_i^3p_j$
*A*_*i*_ *A*_*j*_ *A*_*i*_ *A*_*i*_	0	0	0	0	*p*_*i*_ *p*_*j*_	$2p_i^2p_j$	0	$p_i^2p_j$	$2p_i^3p_j$
*A*_*i*_ *A*_*j*_ *A*_*i*_ *A*_*j*_	0	0	0	0	0	0	2*p*_*i*_*p*_*j*_	*p*_*i*_ *p*_*j*_	$4p_i^2p_j^2$

We here model the uncertainty of the sequencing data through the genotype likelihoods but assume knowledge of population allele frequencies. In the presence of called genotypes (genotypes without uncertainty), our model coincides completely with the approach in Anderson and Weir [8]. In the absence of inbreeding our model reduces to the work in Korneliussen and Moltke [11]. We assume that sites are independent; if they are linked, our likelihood becomes a composite likelihood that will still have consistent estimates even though it has been shown that it can cause relationships to be overestimated [14,15].

This novel method assumes that populations allele frequencies are obtainable, and we note that it has been shown by Csűrös [16] that working in a context of solely diallelic markers, the estimation of the 9 condensed Jaquard coefficients can display an issue of non-identifiability. This will have an impact for some of the summary statistics that are defined as linear combinations of these coefficients, with the estimators that are invariant being *R*, *F*_*a*_, *F*_*b*_, θ, 2 − 3 − IBD, *F*_diff_. Finally ngsRelateV2 also computes 3 summary statistics (last 3 rows of Table 2) based on the 2D-SFS [17], but note that summary statistics based on the 2D-SFS do not require known population allele frequencies—they assume the individuals to be non-inbred. The 2D-SFS obtained in ngsRelateV2 follows the methodology from Korneliussen et al. [18] that is based on genotype likelihoods and therefore does not require called genotypes.

**Table 2:** Various relatedness statistics estimated by ngsRelateV2 and the summary statistics on which they are based

Statistic	Formula	Summary statistic	Source
*r*_*ab*_	*J*_1_ + *J*_7_ + 0.75(*J*_3_ + *J*_5_) + 0.5*J*_8_	IBD	[12]
*F*_*a*_	*J*_1_ + *J*_2_ + *J*_3_ + *J*_4_	IBD	[19]
*F*_*b*_	*J*_1_ + *J*_2_ + *J*_5_ + *J*_6_	IBD	[19]
θ	*J*_1_ + 0.5(*J*_3_ + *J*_5_ + *J*_7_) + 0.25*J*_8_	IBD	[19]
*F*_12_	*J*_1_ + 0.5*J*_3_	IBD	[12]
*F*_21_	*J*_1_ + 0.5*J*_5_	IBD	[12]
Fraternity	*J*_2_ + *J*_7_	IBD	[20]
Identity	*J*_1_	IBD	[20]
Zygosity	*J*_1_ + *J*_2_ + *J*_7_	IBD	[20]
2-3-IBD	*J*_1_ + *J*_2_ + *J*_3_ + *J*_5_ + *J*_7_ + 0.5(*J*_4_ + *J*_6_ + *J*_8_)	IBD	[16]
*F*_diff_	0.5(*J*_4_ − *J*_6_)	IBD	[16]
*R*_0_	(*C* + *G*)/*E*	IBS	[17]
*R*_1_	*E*/(*B* + *D* + *H* + *F* + *C* + *G*)	IBS	[17]
King	[*E* − 2(*C* + *G*)]/(*B* + *D* + *H* + *F* + 2*E*)	IBS	[17]

IBD: identity by descent; IBS: identity by state.

In addition to the raw statistics we have also developed a bootstrapping approach that can be used to recover confidence intervals of all the summary statistics presented in Table 2.

## Simulations

To simulate data with *L* sites and *N* diploid individuals, we first sampled *L* allele frequencies from a uniform distribution with a minor-allele frequency (MAF) filter on 0.05 and 0.1. For each site for each of the *N* individuals, we sample 2 alleles using indepedent Bernoulli trials with the probability of success equal to the allele frequency for the given site, implying that the data are generated under the assumption of Hardy-Weinberg equilibrium. The outcome of these 2 trials represents the genotype. Gametes of these individuals are subsequently generated by sampling either of the 2 alleles from the 2 haplotypes for every site with equal probability. We assume that each site is independent; thus, linkage disequilibrium (LD) is not modeled. Allosomes are disregarded as well.

From the *N* founder individuals, we simulate offspring to generate 3 different pedigrees. From these pedigrees, we have analyzed pairs of individuals with the expected Jacquard coefficients as presented in Table 3.

**Table 3:** Expected Jacquard coefficients, relatedness, and inbreeding coefficients for 3 simulated scenarios

Scenario	*J*_1_	*J*_2_	*J*_3_	*J*_4_	*J*_5_	*J*_6_	*J*_7_	*J*_8_	*J*_9_	*R*	*F*_1_	*F*_2_
1	0	0	0	0	0	0	0	0.25	0.75	0.13	0	0
2	0	0	0	0	0.06	0.19	0	0.38	0.38	0.23	0	0.25
3	0.02	0.02	0.09	0.12	0.06	0.06	0.06	0.38	0.22	0.38	0.25	0.13

We then proceed by calculating genotype likelihoods by assuming different sequencing depths *d* = {1×, 2×, 4×, 8×, 16×}, error rate *e* = 0.001, and number of sites *s* = {10,000, 30,000, 50,000} for the individuals of interest. The per-site-per-individual sequencing depth is given by sampling the depth from a Poisson distribution with parameter *d* and using the binomial density distribution with *e*. This approach is similar to the previous approach in Korneliussen and Moltke [11], which does not model the spatial properties of true recombination and LD.

## Results

**Figure 1:**
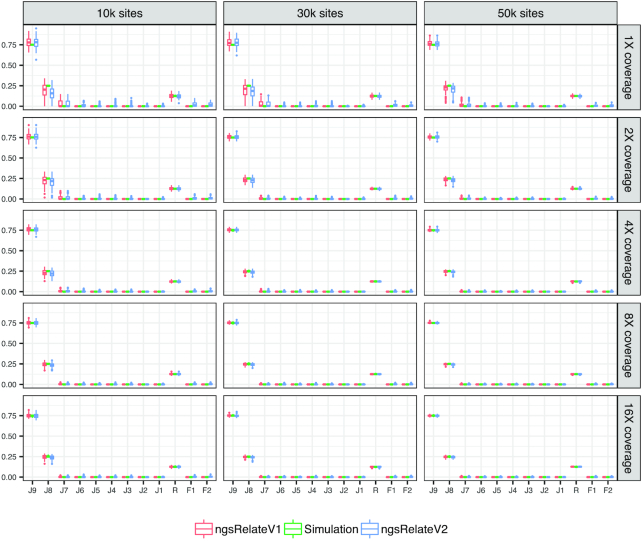
Scenario 1: 100 independent simulations of 2 outbred cousins across variable sequencing depth and informative sites with a MAF cutoff on 10%. *J*_9_ to *J*_1_ refer to the 9 Jacquard coefficients, *R* is the relatedness, and *F*_1_ and *F*_2_ refer to the individual inbreeding coefficients. Simulation (green) are the true values against which we compare ngsRelateV1 (red) and the new program ngsRelateV2 (blue).

**Figure 2:**
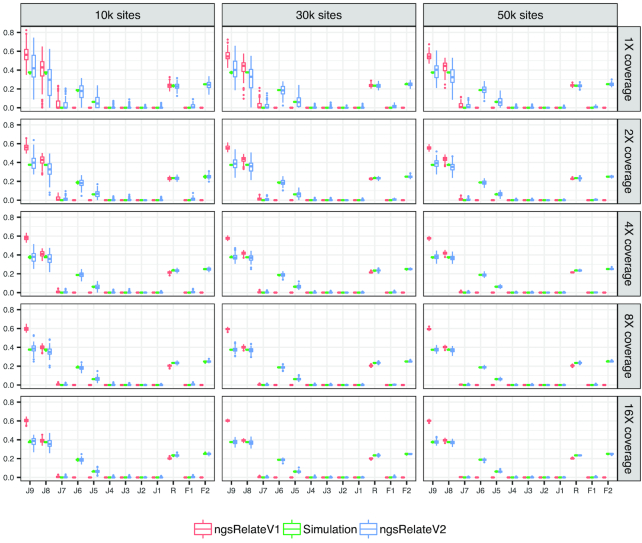
Scenario 2: 100 independent simulations of 2 cousins, with 1 individual being inbred, across variable sequencing depth and segregating sites with a MAF cutoff on 10%. *J*_9_ to *J*_1_ refer to the 9 Jacquard coefficients, *R* is the relatedness, and *F*_1_ and *F*_2_ refer to the individual inbreeding coefficients. Simulation (green) are the true values against which we compare ngsRelateV1 (red) and the new program ngsRelateV2 (blue).

**Figure 3:**
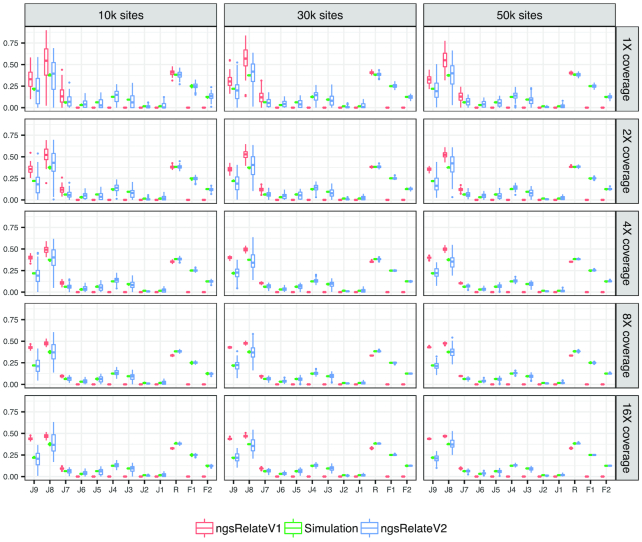
Scenario 3: 100 independent simulations of 2 cousins, both being inbred, across variable sequencing depth and segregating sites with a MAF cutoff on 10%. *J*_9_ to *J*_1_ refer to the 9 Jacquard coefficients, *R* is the relatedness, and *F*_1_ and *F*_2_ refer to the individual inbreeding coefficients. Simulation (green) are the true values against which we compare ngsRelateV1 (red) and the new program ngsRelateV2 (blue).

To test the performance of ngsRelateV2, we use 3 simulated scenarios (see Simulations section) and compare it with ngsRelateV1 [11]. For every scenario, we generate 100 independent simulations for every combination of sequencing effort and number of segregating sites. In Scenario 1, we compare 2 outbred cousins (Fig. 1). As expected, both versions of ngsRelate find not only the correct level of relatedness but also the correct estimates of the 3 relevant Jacquard coefficients (*J*_7_, *J*_8_, *J*_9_). Scenario 2 also includes 2 cousins, but this time we have introduced inbreeding in 1 of the individuals. The parents of the inbred individual are related equivalent to a parent-child relation. In this scenario, even at low sequencing effort and only 10,000 sites, ngsRelateV2 correctly estimates the coefficients of relatedness and inbreeding; however, the estimates of the 9 Jacquard coefficients are somewhat noisy, and ≥50,000 segregating sites are needed to increase the accuracy (Fig. 2). Scenario 3, being the most complex, includes the inbred individual from Scenario 2 and another inbred cousin whose parents are related equivalent to a grandparent-grandchild relationship. Interestingly, with such a complex pedigree, ngsRelateV2 still manages to recover the exact estimates for relatedness and individual inbreeding coefficients, even with only 10,000 segregating sites and a low sequencing depth (Fig. 3). Similarly to the results from Scenario 2, accurate estimates of the 9 Jacquard coefficients required increasing the number of informative sites and/or the sequencing effort. We also applied ngsRelateV2 to these 3 scenarios using a MAF cutoff on 0.05 (Supplementary Figs 1–3). We find that ngsRelateV2 recovers comparable accuracy with a MAF filter on 0.05.

**Figure 4:**
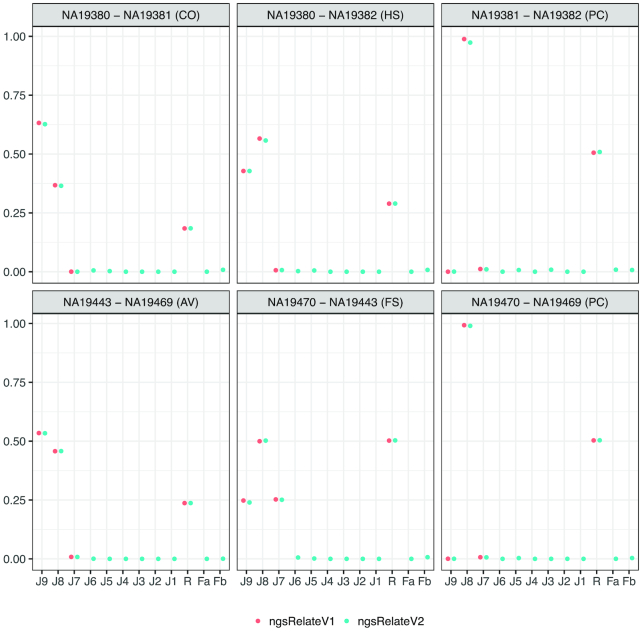
Estimated Jacquard coefficients from 6 pairs of related individuals. The estimates are based on low-depth next-generation sequencing data from the 1000 Genomes Project using ngsRelateV1 and ngsRelateV2. *J*_9_ to *J*_1_ refer to the 9 Jacquard coefficients, *R* is the relatedness, and *F*_1_ and *F*_2_ refer to the individual inbreeding coefficients. CO: cousins; HS: half siblings; PC: parent-child; AV: avuncular; FS: full siblings.

We also applied ngsRelateV2 to real HTS data and compared the estimates with those obtained with ngsRelateV1. We used 6 pairwise related genomes, sequenced to low coverage (∼4×), from the Luhya in Webuye, Kenya (LWK), population generated as part of the 1000 Genomes Project [21]. We calculated genotype likelihoods of the related individuals, using ANGSD [18], at genomic sites with MAF in the LWK population on 0.05, summing up to 4.6 million segregating sites. We not only show that ngsRelateV2 obtains relatedness estimates comparable to those obtained by ngsRelateV1, with this novel software, we also show that all the tested individuals show an inbreeding coefficient <1% (Fig. 4).

In extremely complicated pedigrees with symmetric inbreeding, such as multiple generations of full sibling mating, we find multiple global maxima where several combinations of the 9 Jacquard coefficients, including the expected coefficients, are equally likely. Albeit observing such identifiability challenges, we, importantly, still find accurate relatedness estimates and individual inbreeding coefficients by summing the relevant Jacquard coefficients.

For every pair of individuals, ngsRelateV2 generates and outputs estimates of the 9 Jacquard coefficients, the relatedness, the individual inbreeding coefficients as described above but also other combinations of the 9 Jacquard coefficients: the kinship coefficient, fraternity, and the 3 summary statistics inbred relatedness, identity, and zygosity, suggested by Ackerman and colleagues [20]. It also produces the King statistic [22] based on the 2D site frequency spectrum of pairs of individuals following the methodology of Waples et al. [17]. The latter statistics do not require population allele frequencies.

### Computational speed and memory requirements

**Figure 5:**
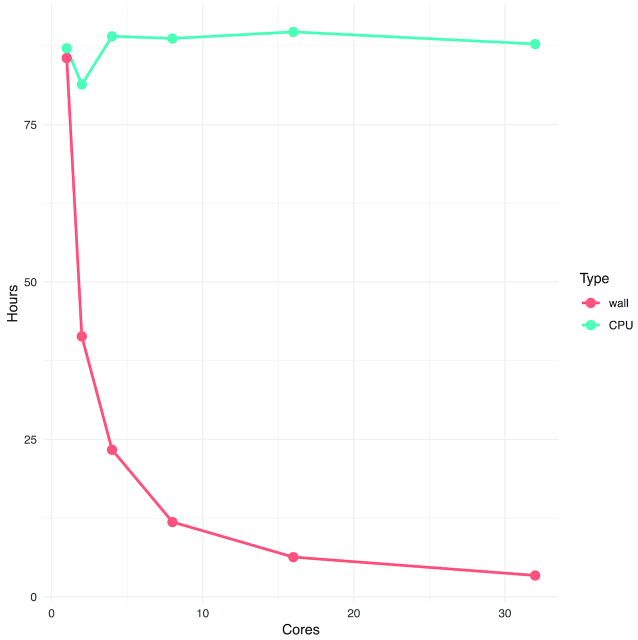
Runtimes for ngsRelateV2 on a dataset with 135 individuals with 164 mio possible single-nucleotide polymorphism sites. The blue line indicates the overall CPU usage across all threads allocated to the main process. The red line indicates the runtime for the process to finish. The actual values along with memory usage can be found in Table 4.

To take advantage of the increasing number of cores of available on modern computers we employ a multilevel threading approach by parallelizing both the file reading and the actual analysis. In Fig. 5 we analyzed a semi-random dataset consisting of 135 samples mainly from de Barros et al. [23]. The input for the program was a 34-GB BCF file generated with standard bcftools with a liberal 164 million number of single-nucleotide polymorphism sites. We timed the actual runtime (wall clock) and the CPU time for a varying number of cores (1, 2, 4, 8, 16, 32) and noted the memory usage for each run because allocating more cores for the process requires additional internal datastructures and therefore also increases the memory requirements as reported in Table 4. From both the table and figure we observe a near linear correlation between the number of cores and the runtime, with the CPU time remaining almost constant.

**Table 4:** Run statistics for 34-GB BCF file as a function of number of cores

Cores	Memory usage (GB)	Wall clock time (h)	CPU time (h)
1	45	85.59	87.14
2	46.9	41.38	81.39
4	49.3	23.36	89.03
8	54.2	11.88	88.69
16	63.5	6.30	89.73
32	83.2	3.39	87.81

Presented are the memory requirement, wall clock time (actual runtime), and CPU time; see also Fig. 5.

## Conclusion

The tool presented in this Technical Note allows researchers to perform relatedness analysis for inbred individuals in a statistical framework that is especially suited for low-coverage sequence data. The results show that the method performs well for estimating all 9 coefficients, at least when the underlying pedigrees are not extremely complex. And even when the underlying pedigree is very complex, compound summaries of the output, such as relatedness and inbreeding coefficients, will still be correct. The implementation is a fast multi-threaded C++ program that can be directly applied to the most commonly used data files used for HTS data.

## Implementation Details

The program is implemented in a fast multi-threaded C++ program and takes as input either genotype likelihood files and frequencies, BCF/VCF files as produced from standard tools such as GATK [24] or SAMtools [25], or binary-format PLINK files [3]. We also include an R implementation that we used for simulating data. Of note, the simulations generated in this study do not account for LD. In case of LD between genetic variants, the likelihood function becomes a composite likelihood function. The maximum-likelihood estimate of such a function is consistent with that found with a likelihood function of independent sites [26].

The optimization follows the approach described in Korneliussen and Moltke [11]. The optimization is an accelerated expectation maximization following the squared iterative approach in **S3** in Varadhan and Roland [27] and is initialized with a random start point within the parameter space. The borders of the parameter space are manually examined after convergence. Because the expectation maximization algorithm is only guaranteed to find a local optimum, it is recommended to rerun with multiple different seeds although we note that we did not find an issue with multiple local optima in our examples.

## Supplementary Material

GIGA-D-18-00338_Original-Submission.pdf

GIGA-D-18-00338_Revision-1.pdf

GIGA-D-18-00338_Revision-2.pdf

Response-to-Reviewer-Comments-Revision-1.pdf

Response-to-Reviewer-Comments_Original-Submission.pdf

Reviewer-1-Report-Original-Submission -- Jinliang Wang 9/23/2018 Reviewed

Reviewer-2-Report-Original-Submission -- Jan Graffelman 10/8/2018 Reviewed

Reviewer-2-Report-Revision-1 -- Jan Graffelman 1/15/2019 Reviewed

Supplement_Figures.pdf

## Availability of source code and requirements

- Project name: ngsRelateV2
- Project home page: http://github.com/ANGSD/ngsRelate
- Operating system(s): platform independent
- Programming language: C++
- Other requirements: htslib (only for parsing VCF/BCF files)
- License: GNU GPL (version 3)
- RRID: SCR_016588
- GigaDB: Snapshots of the code and other supporting data are available in the *GigaScience* repository [28]

## Additional files

Supplementary Methods and Results are available via the additional file associated with this article.

## Abbreviations

HTS: high-throughput sequencing; IBD: identity by descent; IBS: identity by state; LD: linkage disequilibrium; LWK: Luhya in Webuye, Kenya; MAF: minor-allele frequency.
