# Supplementary material for: Fast and accurate relatedness estimation from high-throughput sequencing data in the presence of inbreeding
Source: Gigascience. 2019 Apr 30;8(5):giz034. doi: 10.1093/gigascience/giz034 (PMC6488770; doi:10.1093/gigascience/giz034)
Supplement: GIGA-D-18-00338_Original-Submission.pdf [file giz034_giga-d-18-00338_original-submission.pdf]

GigaScience

Fast and accurate relatedness estimation from high throughput sequencing data in the presence of inbreeding

--Manuscript Draft--

**Manuscript Number:**	GIGA-D-18-00338
**Full Title:**	Fast and accurate relatedness estimation from high throughput sequencing data in the presence of inbreeding
**Article Type:**	Technical Note
**Funding Information:**	Carlsbergfondet (DK) (CF16-0913) — Dr Thorfinn Sand Korneliussen<br>Danmarks Grundforskningsfond (DK) (DNRF94) — Mr Kristian Hanghoej<br>initiative d'Excellence Chaires d'attractivité (OURASI) — Mr Kristian Hanghoej<br>Ydun (NA) — Dr Ida Moltke<br>ERC consolidator grant (LocalAdaptation 647787) — Dr Andrea Manica
**Abstract:**	The estimation of relatedness between pairs of possibly inbred individuals from high-throughput sequencing (HTS) data has previously not been possible for samples where we can not obtain reliable genotype calls, as in the case low coverage data. Results: We introduce ngsRelateV2, a major revision of ngsRelateV1; a program which originally allowed for estimation of relatedness from HTS data among uninbred individuals only. The new revised version takes into account the possibility of individuals being inbred by estimating the nine condensed Jacquard coefficients along with various other relatedness statistics. The program is threaded and scales linearly with the number of cores allocated to the process.<br>Conclusion: The program is available as an open source c/c++ program under the GPL license and hosted at https://github.com/ANGSD/ngsRelate. To facilitate easy analysis, the program is able to work directly on the most commonly used container formats for raw sequence (BAM/CRAM) and summary data (VCF/BCF).
**Corresponding Author:**	Thorfinn Sand Korneliussen, Ph.D<br>Natural History Museum of Denmark<br>Copenhagen, DENMARK
**Corresponding Author Secondary Information:**	
**Corresponding Author's Institution:**	Natural History Museum of Denmark
**Corresponding Author's Secondary Institution:**	
**First Author:**	Kristian Hanghoej, msc
**First Author Secondary Information:**	
**Order of Authors:**	Kristian Hanghoej, msc<br>Ida Moltke, PhD<br>Andrea Manica, PhD<br>Thorfinn Sand Korneliussen, Ph.D
**Order of Authors Secondary Information:**	
**Additional Information:**	
**Question**	**Response**
Are you submitting this manuscript to a special series or article collection?	No

Powered by Editorial Manager® and ProduXion Manager® from Aries Systems Corporation

**Experimental design and statistics**<br><br>Full details of the experimental design and statistical methods used should be given in the Methods section, as detailed in our Minimum Standards Reporting Checklist. Information essential to interpreting the data presented should be made available in the figure legends.<br><br>Have you included all the information requested in your manuscript?	Yes
**Resources**<br><br>A description of all resources used, including antibodies, cell lines, animals and software tools, with enough information to allow them to be uniquely identified, should be included in the Methods section. Authors are strongly encouraged to cite Research Resource Identifiers (RRIDs) for antibodies, model organisms and tools, where possible.<br><br>Have you included the information requested as detailed in our Minimum Standards Reporting Checklist?	Yes
**Availability of data and materials**<br><br>All datasets and code on which the conclusions of the paper rely must be either included in your submission or deposited in publicly available repositories (where available and ethically appropriate), referencing such data using a unique identifier in the references and in the "Availability of Data and Materials" section of your manuscript.<br><br>Have you have met the above requirement as detailed in our Minimum Standards Reporting Checklist?	Yes

Manuscript Click here to download Manuscript ibd-gigascience-9.pdf

GigaScience, 2018, 1–6

doi: xx.xxxx/xxxx
Manuscript in Preparation
Technical Note

TECHNICAL NOTE

Fast and accurate relatedness estimation from high throughput sequencing data in the presence of inbreeding

Kristian Hanghøj[1,2,*], Ida Moltke[3], Andrea Manica[4] and Thorfinn Sand Korneliussen[1,4,*]

[1]Centre for GeoGenetics, Natural History Museum of Denmark, University of Copenhagen, 1350 Copenhagen K, Denmark and [2]Université de Toulouse, University Paul Sabatier (UPS), Laboratoire AMIS, CNRS UMR 5288, Toulouse, France and [3]Department of Biology, University of Copenhagen, Denmark and [4]Department of Zoology, University of Cambridge, Downing Street, Cambridge CB2 3EJ, UK.

[*]k.hanghoej@snm.ku.dk; tskorneliussen@snm.ku.dk

Abstract

Background: The estimation of relatedness between pairs of possibly inbred individuals from high-throughput sequencing (HTS) data has previously not been possible for samples where we can not obtain reliable genotype calls, as in the case low coverage data.
Results: We introduce ngsRelateV2, a major revision of ngsRelateV1; a program which originally allowed for estimation of relatedness from HTS data among uninbred individuals only. The new revised version takes into account the possibility of individuals being inbred by estimating the nine condensed Jacquard coefficients along with various other relatedness statistics. The program is threaded and scales linearly with the number of cores allocated to the process.
Conclusion: The program is available as an open source c/c++ program under the GPL license and hosted at https://github.com/ANGSD/ngsRelate. To facilitate easy analysis, the program is able to work directly on the most commonly used container formats for raw sequence (BAM/CRAM) and summary data (VCF/BCF).
Supplementary information: None

Key words: Relatedness estimation; inbreeding; Jacquard coefficients; high throughput sequencing data; genotype likelihood; NGS;threading.

Introduction

Being able to estimate how related two individuals are and whether they are inbred is important in several different fields ranging from conservation genetics to medical genetics. For this purpose, numerous coefficients, like the kinship coefficient and inbreeding coefficients, have been defined and many programs for estimating these coefficients have been proposed.

Notably, the genetic relationship between two individuals can be quantified by the extent to which the two individuals share their alleles identical-by-descent (IBD); i.e. are identical due to recent common ancestry. More specifically, for two diploid individuals, and thus four alleles, there are 15 distinct possible IBD sharing patterns at any given site (detailed identity states). If we ignore the maternal or paternal origin of the alleles, these 15 detailed states can be collapsed into nine condensed states [1] (here denoted $j_1, j_2, \ldots, j_9$), and their corresponding frequency in the genome of two individuals are called the condensed Jacquard coefficients (here denoted $\mathbf{J_1}$,$\mathbf{J_2}$,...,$\mathbf{J_9}$). These condensed coefficients provide a comprehensive description of the common ancestry between two individuals, that can

Compiled on: September 1, 2018.
Draft manuscript prepared by the author.

be used to infer their familial relationship. Furthermore, many other commonly used coefficients, such as the kinship coefficient and inbreeding coefficients, can be expressed as linear combinations of the nine condensed Jacquard coefficients.

In the specific case where neither individual is inbred, only three of the condensed Jacquard coefficients can be positive, namely $\mathbf{J_7}$,$\mathbf{J_8}$ and $\mathbf{J_9}$, which are often also denoted $\mathbf{k_2}$,$\mathbf{k_1}$ and $\mathbf{k_0}$, respectively. Numerous approaches, based on either method of moments (e.g. [2]) or maximum-likelihood estimation (e.g. [3]), have been devised to estimate these three quantities assuming that the rest are zero and thus that the individuals are not inbred. This includes commonly used methods like PLINK and KING [2, 4]. Importantly, these methods can lead to wrong estimates and conclusions if applied to inbred individuals because the assumption that only J_7, J_8, and J_9 can be positive is violated. Hence in the presence of inbreeding one needs to estimate all nine coefficients. Several methods for doing this have been proposed [5, 6, 7]. However, very few current tools allow the user to do this and the few that do all require high quality genotype data as input (e.g. [7]). They can therefore not be applied to HTS data of low depth, which is sometimes the only data available. Until recently the same was the case for all the methods for estimating relatedness between non-inbred individual. E.g. both PLINK and KING only work for genotype data. However, recently a few methods that can be applied to low depth sequencing data have been developed [8, 9]. One of these is ngsRelate [9] (hereafter referred to as ngsRelateV1), which works by integrating over every possible genotypic configurations and assigning these a probability given by their genotype likelihood. We here extend this software (hereafter referred to as ngsRelateV2) so it allows the user to infer all nine Jacquard coefficients, and thus allow for inference of relatedness in the presence of inbreeding as well as inbreeding coefficient for both individuals.

Materials & Methods

The underlying statistical framework is similar to that from ngsRelateV1 [9]. Given two individuals, i and j, from the same homogeneous population, we let D_l^i and D_l^j denote the observed HTS data at a biallelic locus l, and G_l^i and G_l^j denote the true, unobserved genotypes at the same locus. Furthermore, we let f_l denote the allele frequency at locus l in the relevant population and X_l denote the unobserved IBD state of the two individuals at locus l. Using this notation we can write the likelihood of the condensed Jacquard coefficients, $\mathbf{J} = (J_1, J_2, J_3, J_4, J_5, J_6, J_7, J_8, J_9)$, for L independent (i.e.unlinked) biallelic loci as:

$$L(J|D^i, D^j, f^A) = \prod_{l=1}^{L} \sum_{m \in \mathbf{J}} P(D_l^i, D_l^j \mid X_l = m, f_l^A) P(X_l = m|J),$$

Notably, here $P(X_l = m \mid J) = J_m$ and $P(D_l^i, D_l^j \mid X_l = m, f_l^A)$ can be rewritten as follows:

$$P(D_l^i, D_l^j \mid X_l = m, f_l^A)$$

$$= \sum_{G_l^i, G_l^j \in \{0,1,2\}^2} P(D_l^i \mid G_l^i) P(D_l^j \mid G_l^j) P(G_l^i, G_l^j \mid f_l^A, X_l = m).$$

where $P(D_l^i|G_l^i)$ and $P(D_l^j|G_l^j)$ denote the per individual genotypes likelihoods for a biallelic locus l, which can be calculated from the sequencing data and $P(G_l^i, G_l^j \mid f_l^A)$ is given from Table 1. We use this likelihood function as a basis for performing maximum likelihood estimation. A number of useful estimates can be calculated directly from J, such as relatedness ($R = J_1 + J_7 + \frac{3}{4}(J_3 + J_5) + \frac{1}{2}J_8$), defined as the proportion of homologous alleles IDB [10], and per individual inbreeding coefficients, F_1 ans F_2 (as in [11]).

We note that we here model the uncertainty of the sequencing data through the genotype likelihoods, but assume knowledge of population frequencies. In the presence of called genotypes (genotypes without uncertainty), our model coincides completely with the approach in [7]. In the absence of inbreeding our model reduces to the work in [9]. We assume that sites are independent, if they are linked our likelihood becomes a composite likelihood that will still have consistent estimates even though it has been shown that it can cause relationships to be overestimated [12, 13].

We note that we assume that correct population allele frequencies are obtainable, which is in contrast with other recent approaches [14, 8]. However, those methods do not allow for inbreeding.

Simulations

To simulate data with L sites and N diploid individuals, we first sampled L allele frequencies from a uniform distribution with a minor allele frequency filter on 0.1. For each site for each of the N individuals, we sample two alleles using Bernoulli trials with the probability of success equal to the allele frequency for the given site. The outcome of these two trials represent the genotype. Gametes of these individuals are subsequently generated by sampling either of the two alleles from the two haplotypes for every site with equal probability. We assume that each site is independent, thus, linkage disequilibrium is not modeled. Allosomes are disregarded as well.

From the N founder individuals, we simulate offspring to generate three different pedigrees. From these pedigrees, we have analyzed pairs of individuals with the expected Jacquard coefficients as shown in Table 2.

We then proceed by calculating genotype likelihoods by assuming different sequencing depths $d = \{2X, 4X, 8X, 16X\}$, error rate $e = 0.001$ and number of sites $s = \{10K, 30K, 50K\}$ for the individuals of interest. The per-site-per-individual sequencing depth is given by sampling the depth from a Poisson distribution with parameter d and using the binomial density distribution with e.

Results

To test the performance of ngsRelateV2, we use three simulated scenarios (see Simulations section) and compare it to ngsRelateV1 [9]. For every scenario, we generate 100 independent simulations for every combination of sequencing effort and number of segregating sites. In the first scenario, we compare two out-bred cousins (fig.1). As expected, both version of ngsRelate find not only the correct level of relatedness, but also the correct estimates of the three relevant Jacquard coefficients (J_7, J_8, J_9). The second scenario also includes two cousins, but this time we have introduced inbreeding in one of the individuals. The parents of the inbred individual are related corresponding to a parent-child relation. In this scenario, even at low sequencing effort and 10k sites, ngsRelateV2 correctly estimates the coefficients of relatedness and inbreeding; however, the estimates of the nine Jacquard coefficients are somewhat noisy, and at least 50k segregating sites are needed to reduce the confidence intervals (fig.2). The final scenario, being the

Table 1. Probabilities for various allelic states, given modes of IDB from Table 1 in [7]. Triallelic sites are disregarded.

Allelic State	J_1	J_2	J_3	J_4	J_5	J_6	J_7	J_8	J_9
$A_iA_iA_iA_i$	p_i	p_i^2	p_i^2	p_i^3	p_i^2	p_i^3	p_i^2	p_i^3	p_i^4
$A_iA_iA_jA_j$	0	p_ip_j	0	p_ip_j	0	$p_i^2p_j$	0	0	$p_i^2p_j^2$
$A_iA_iA_iA_j$	0	0	p_ip_j	$2p_i^2p_j$	0	0	0	$p_i^2p_j$	$2p_i^3p_j$
$A_iA_jA_iA_i$	0	0	0	0	p_ip_j	$2p_i^2p_j$	0	$p_i^2p_j$	$2p_i^3p_j$
$A_iA_jA_iA_j$	0	0	0	0	0	0	$2p_ip_j$	p_ip_j	$4p_i^2p_j^2$

Table 2. Expected Jacquard coefficients, relatedness and inbreeding coefficients for three simulated scenarios.

	J_1	J_2	J_3	J_4	J_5	J_6	J_7	J_8	J_9	R	F_1	F_2
scenario_1	0	0	0	0	0	0	0	0.25	0.75	0.13	0	0
scenario_2	0	0	0	0	0.06	0.19	0	0.38	0.38	0.23	0	0.25
scenario_3	0.02	0.02	0.09	0.12	0.06	0.06	0.06	0.38	0.22	0.38	0.25	0.13

most complex, includes the inbred individual from scenario two and another inbred cousin with its parents being related corresponding to a grandparent-grandchild relation. Interestingly, with such a complex pedigree, ngsRelateV2 still manages to recover the exact estimates for relatedness and individual inbreeding coefficients, even with only 10k segregating sites and a low sequencing depth (fig.3). Similarly to the results from scenario two, confident estimates of the nine Jacquard coefficients required increasing the number of informative sites and/or the sequencing effort.

In extremely complicated pedigrees with symmetric inbreeding, such as multiple generations of full sibling mating, we find multiple global maxima where several combinations of the nine Jacquard coefficients, including the expected coefficients, are equally likely. Albeit observing such identifiability challenges, we, importantly, still find accurate relatedness estimates and individual inbreeding coefficients by summing the relevant Jacquard coefficients.

For every pair of individuals, ngsRelateV2 generates and outputs estimates of the nine Jacquard coefficients, the relatedness, the individual inbreeding coefficients as described above but also other combinations of the nine Jacquard coefficient: the kinship coefficient, fraternity, and the three summary statistics inbred relatedness, identity, and zygosity, suggested by Ackerman and colleagues [15]. Thanks to threading, ngsRelateV2 scales well to large numbers of pairwise comparisons of individuals.

Conclusion

The tool presented in this technical note allows researchers to perform relatedness analysis for inbred individuals in a statistical framework that is especially suited for low coverage sequence data. The results show that the method performs well for estimating all nine coefficients, at least when the underlying pedigrees are not extremely complex. And even when the underlying pedigree is extremely complex, compound summaries of the output, like relatedness and inbreeding coefficients, will still be correct. The implementation is a fast multi threaded c++ program that can be directly applied to the most commonly used data files used for high throughput sequencing data.

Implementation Details

The program is implemented in a fast multithreaded c++ program and takes as input either genotype likelihood files and frequencies or bcf/vcf files as produced from standard tools such as GATK[16] or SAMtools [17]. We also include an R implementation that we used for prototyping for early versions of the program together with our simulation program.

Availability of source code and requirements

- Project name: ngsRelateV2
- Project home page: http://github.com/ANGSD/ngsRelate
- Operating system(s): Platform independent
- Programming language: C++
- Other requirements: htslib (only for parsing VCF/BCF files)
- License: GNU GPL and FreeBSD and public domain etc.

Declarations

List of abbreviations

IBD: Identity-by-descent. HTS: High-throughput sequencing.

Consent for publication

Not applicable.

Competing Interests

None.

Funding

KH is funded by the Danish National Research Foundation (DNRF94) and the Initiative d'Excellence Chaires d'attractivité, Université de Toulouse (OURASI); TSK by a grant from the Carlsberg Foundation (CF16-0913); IM by Ydun; AM by an ERC Consolidator Grant LocalAdaptation 647787.

Authors' Contributions

KH implemented and ran all analyses. IM and AM devised scenarios and improved early versions of the method. TSK devised the model. All authors wrote the article.

Acknowledgements

The program was first prototyped by Philip Alstrup Andersen, a Master student under the supervision of TSK and IM.

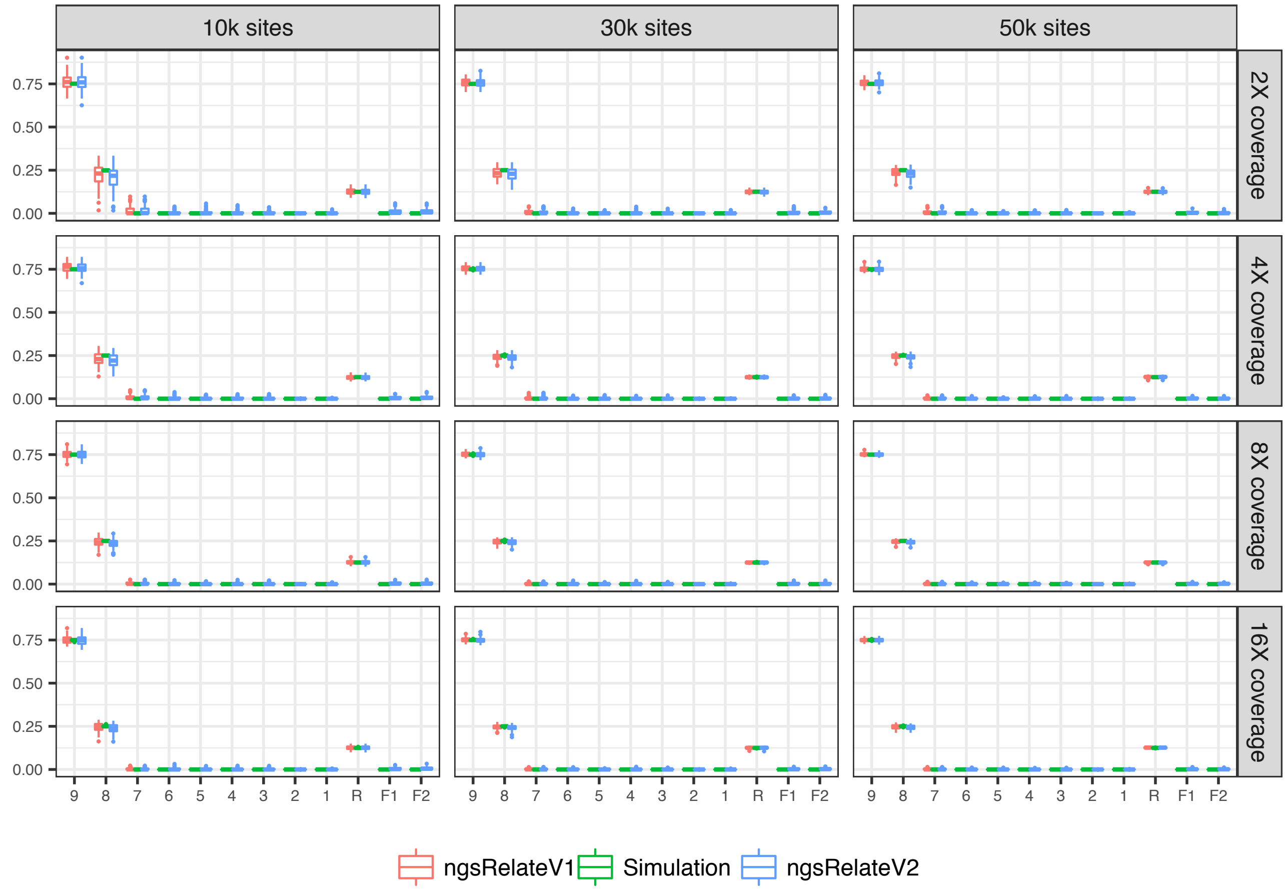

Figure 1. 100 independent simulations of two out-bred cousins across variable sequencing depth and informative sites. J_9 to J_1 refer to the nine Jacquard coefficients, R is the relatedness, finally, F_1 and F_2 refer to the individual inbreeding coefficients. Simulation (green) are the true values that we compare ngsRelateV1 (red) and the new program ngsRelateV2 (blue) against.

References

1. Weir BS, Anderson AD, Hepler AB. Genetic relatedness analysis: modern data and new challenges. Nat Rev Genet 2006 Oct;7(10):771–780.
2. Purcell S, Neale B, Todd-Brown K, Thomas L, Ferreira MA, Bender D, et al. PLINK: a tool set for whole-genome association and population-based linkage analyses. Am J Hum Genet 2007 Sep;81(3):559–575.
3. Thompson EA. The estimation of pairwise relationships. Ann Hum Genet 1975 Oct;39(2):173–188.
4. Manichaikul A, Mychaleckyj JC, Rich SS, Daly K, Sale M, Chen WM. Robust relationship inference in genome-wide association studies. Bioinformatics 2010 Nov;26(22):2867–2873.
5. Ritland K. Estimators for pairwise relatedness and individual inbreeding coefficients. Genetical Research 1996;67(2):175–185.
6. Milligan BG. Maximum-likelihood estimation of relatedness. Genetics 2003 Mar;163(3):1153–1167.
7. Anderson AD, Weir BS. A maximum-likelihood method for the estimation of pairwise relatedness in structured populations. Genetics 2007 May;176(1):421–440.
8. Kuhn JMM, Jakobsson M, Gunther T. Estimating genetic kin relationships in prehistoric populations. PLoS ONE 2018;13(4):e0195491.
9. Korneliussen TS, Moltke I. NgsRelate: a software tool for estimating pairwise relatedness from next-generation sequencing data. Bioinformatics 2015 Dec;31(24):4009–4011.
10. Hedrick PW, Lacy RC. Measuring Relatedness between Inbred Individuals. Journal of Heredity 2015;106(1):20–25. http://dx.doi.org/10.1093/jhered/esu072.
11. Vieira FG, Fumagalli M, Albrechtsen A, Nielsen R. Estimating inbreeding coefficients from NGS data: Impact on genotype calling and allele frequency estimation. Genome Res 2013 Nov;23(11):1852–1861.
12. Ko A, Nielsen R. Composite likelihood method for inferring local pedigrees. PLoS Genet 2017 Aug;13(8):e1006963.
13. Sun M, Jobling MA, Taliun D, Pramstaller PP, Egeland T, Sheehan NA. On the use of dense SNP marker data for the identification of distant relative pairs. Theor Popul Biol 2016 Feb;107:14–25.
14. Waples RK, Albrechtsen A, Moltke I. Allele frequency-free inference of close familial relationships from genotypes or low depth sequencing data. bioRxiv 2018;https://www.biorxiv.org/content/early/2018/02/06/260497.
15. Ackerman MS, Johri P, Spitze K, Xu S, Doak TG, Young K, et al. Estimating Seven Coefficients of Pairwise Relatedness Using Population-Genomic Data. Genetics 2017 05;206(1):105–118.
16. McKenna A, Hanna M, Banks E, Sivachenko A, Cibulskis K, Kernytsky A, et al. The Genome Analysis Toolkit: a MapReduce framework for analyzing next-generation DNA sequencing data. Genome Res 2010 Sep;20(9):1297–1303.
17. Li H, Handsaker B, Wysoker A, Fennell T, Ruan J, Homer N, et al. The Sequence Alignment/Map format and SAMtools. Bioinformatics 2009 Aug;25(16):2078–2079.

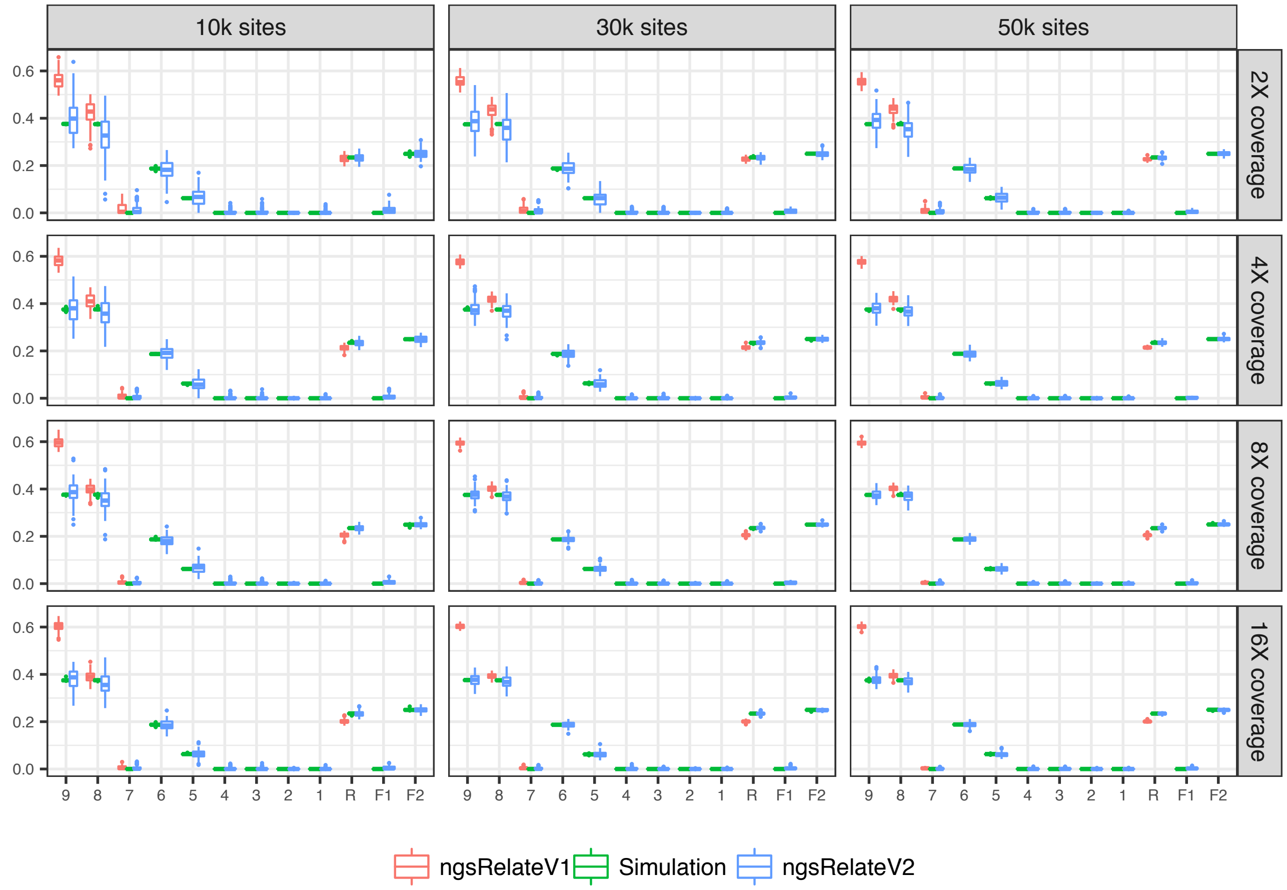

Figure 2. 100 independent simulations of two cousins, with one individual being inbred, across variable sequencing depth and segregating sites. J_9 to J_1 refer to the nine acquard coefficients, R is the relatedness, finally, F_1 and F_2 refer to the individual inbreeding coefficients. Simulation (green) are the true values that we compare ngsRelateV1 (red) and the new program ngsRelateV2 (blue) against.

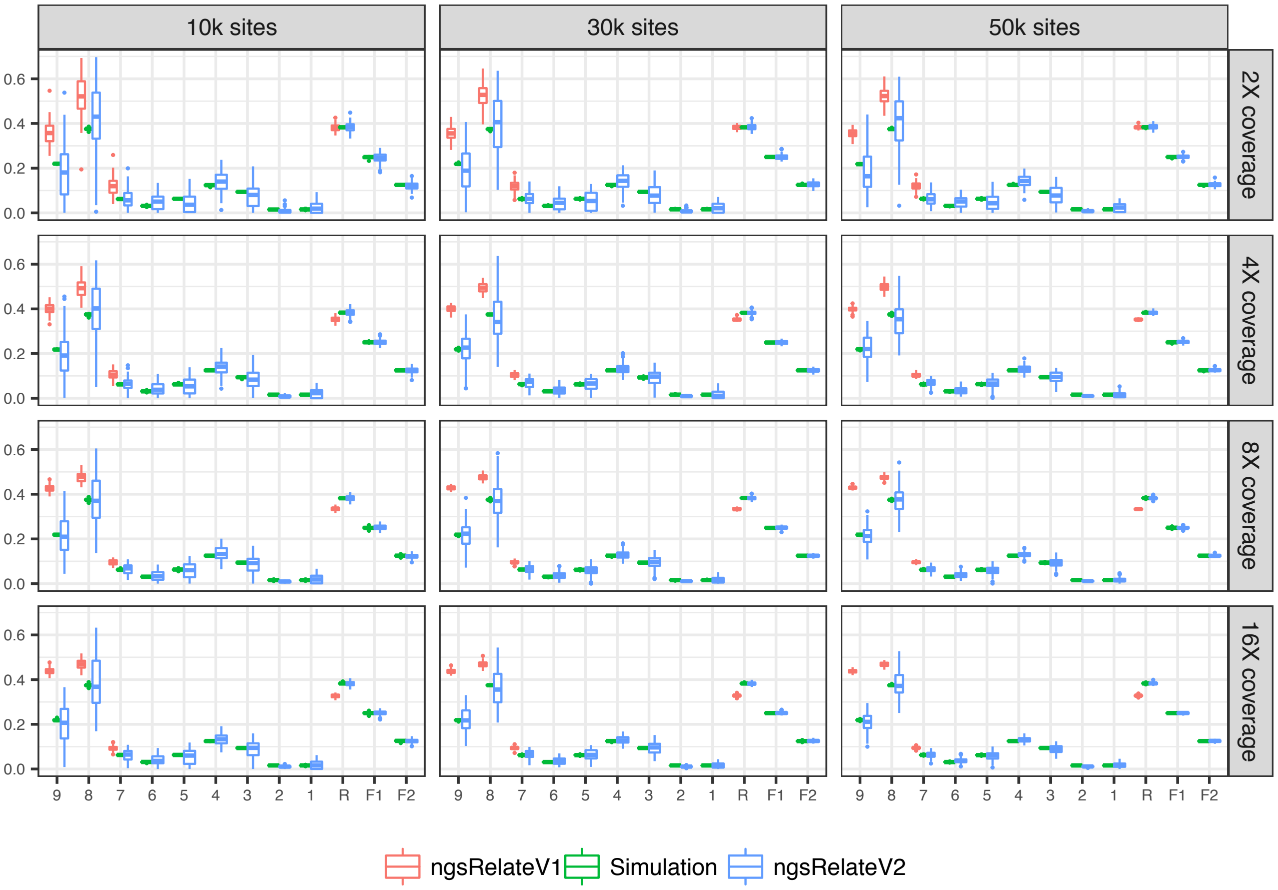

Figure 3. 100 independent simulations of two cousins, both being inbred, across variable sequencing depth and segregating sites. J_9 to J_1 refer to the nine jacquard coefficients, R is the relatedness, finally, F_1 and F_2 refer to the individual inbreeding coefficients. Simulation (green) are the true values that we compare ngsRelateV1 (red) and the new program ngsRelateV2 (blue) against.
