# Supplementary material for: Fast and accurate relatedness estimation from high-throughput sequencing data in the presence of inbreeding
Source: Gigascience. 2019 Apr 30;8(5):giz034. doi: 10.1093/gigascience/giz034 (PMC6488770; doi:10.1093/gigascience/giz034)
Supplement: GIGA-D-18-00338_Revision-1.pdf [file giz034_giga-d-18-00338_revision-1.pdf]

GigaScience

Fast and accurate relatedness estimation from high throughput sequencing data in the presence of inbreeding

--Manuscript Draft--

**Manuscript Number:**	GIGA-D-18-00338R1
**Full Title:**	Fast and accurate relatedness estimation from high throughput sequencing data in the presence of inbreeding
**Article Type:**	Technical Note
**Funding Information:**	Carlsbergfondet (DK) (CF16-0913) — Dr Thorfinn Sand Korneliussen<br>Danmarks Grundforskningsfond (DK) (DNRF94) — Mr Kristian Hanghoej<br>initiative d’Excellence Chaires d’attractivité (OURASI) — Mr Kristian Hanghoej<br>Ydun (NA) — Dr Ida Moltke<br>ERC consolidator grant (LocalAdaptation 647787) — Dr Andrea Manica
**Abstract:**	The estimation of relatedness between pairs of possibly inbred individuals from high-throughput sequencing (HTS) data has previously not been possible for samples where we can not obtain reliable genotype calls, as in the case low coverage data.<br>Results: We introduce ngsRelateV2, a major revision of ngsRelateV1; a program which originally allowed for estimation of relatedness from HTS data among uninbred individuals only. The new revised version takes into account the possibility of individuals being inbred by estimating the nine condensed Jacquard coefficients along with various other relatedness statistics. The program is threaded and scales linearly with the number of cores allocated to the process.<br>Conclusion: The program is available as an open source c/c++ program under the GPL license and hosted at https://github.com/ANGSD/ngsRelate. To facilitate easy analysis, the program is able to work directly on the most commonly used container formats for raw sequence (BAM/CRAM) and summary data (VCF/BCF).
**Corresponding Author:**	Thorfinn Sand Korneliussen, Ph.D<br>Natural History Museum of Denmark<br>Copenhagen, DENMARK
**Corresponding Author Secondary Information:**	
**Corresponding Author's Institution:**	Natural History Museum of Denmark
**Corresponding Author's Secondary Institution:**	
**First Author:**	Kristian Hanghoej, msc
**First Author Secondary Information:**	
**Order of Authors:**	Kristian Hanghoej, msc<br>Ida Moltke, PhD<br>Andrea Manica, PhD<br>Thorfinn Sand Korneliussen, Ph.D
**Order of Authors Secondary Information:**	
**Response to Reviewers:**	GIGA-D-18-00338<br>Fast and accurate relatedness estimation from high throughput sequencing data in the presence of inbreeding<br>Kristian Hanghoej, msc; Ida Moltke, PhD; Andrea Manica, PhD; Thorfinn Sand Korneliussen, Ph.D<br>GigaScience

Powered by Editorial Manager® and ProduXion Manager® from Aries Systems Corporation

Dear Dr Korneliussen,

Your manuscript "Fast and accurate relatedness estimation from high throughput sequencing data in the presence of inbreeding" (GIGA-D-18-00338) has been assessed by our reviewers. Although it is of interest, we are unable to consider it for publication in its current form. The reviewers have raised a number of points which we believe would improve the manuscript and may allow a revised version to be published in GigaScience.

Their reports are below.

Please address all of the reviewers' points, but I'd like to highlight two concerns that are really crucial for the potential acceptance of a revised version:

1. Both reviewers mention that real data should be used to demonstrate the tool's capabilities in addition to your simulations. This recommendation is in line with the expected standard for our "Technical Notes", and I urge you to present a use case with real data.

We agree with the reviewers and added six pairwise comparisons of related individuals. ngsRelateV2 obtains comparable relatedness estimates to those obtained with ngsRelateV1. We also show that ngsRelateV2 in a single run obtains inbreeding coefficient for all the analyzed individuals. See also comment 7 from reviewer 2. We have screened the literature for populations with a pair of related individuals that also show a nonzero level of inbreeding. This includes a rice data set (doi:10.1101/gr.157388.113) and a population of mountain gorillas (DOI:10.1126/science.aaa3952). However, we have not been able to identify a data set, that full fill both of the above-mentioned criteria. If you are aware of such dataset, we will be pleased to apply ngsRelateV2 to it.

2. Related to the first point, reviewer 2 wonders how the tool performs with fewer restrictions, and in a more realistic setting. Please carefully explore and discuss this aspect in the revised manuscript, following the guidance of our reviewers.

As described in our reply to the first reviewers concern (comment 5), we have now performed additional simulations with fewer restrictions. Specifically, as requested by the reviewer, we have performed all simulations based on a minor allele frequency filter on 0.05 as well. These additional simulations demonstrate that ngsRelateV2 also manages to obtain the correct related and inbreeding estimates with a less stringent filtering.

In addition, please register the new software application in the SciCrunch.org database to receive a RRID (Research Resource Identification Initiative ID) number, and include this in your manuscript. This will facilitate tracking, reproducibility and re-use of your tool.
We have now registered the software on scicrunch and have obtained the RRID SCR_016588 which we have included in the manuscript section ‘Availability’.

Please also clarify in the manuscript which open licence is applied for your code (the statement seems ambiguous at the moment).
We have set the licence to GPL v3.

If you are able to fully address these points, we would encourage you to submit a revised manuscript to GigaScience. Once you have made the necessary corrections, please submit online at:

https://giga.editorialmanager.com/

If you have forgotten your username or password please use the "Send Login Details" link to get your login information. For security reasons, your password will be reset.

Please include a point-by-point within the 'Response to Reviewers' box in the submission system. Please ensure you describe additional experiments that were carried out and include a detailed rebuttal of any criticisms or requested revisions that you disagreed with. Please also ensure that your revised manuscript conforms to the journal style, which can be found in the Instructions for Authors on the journal homepage.

The due date for submitting the revised version of your article is 06 Jan 2019.

I look forward to receiving your revised manuscript soon.

Best wishes,

Hans Zauner
GigaScience
www.gigasciencejournal.com

Reviewer reports:

Reviewer #1: In this MS, the authors introduce a new method to estimate the 9 condensed IBD coefficients from high throughput sequencing data (with genotyping errors). It is implemented in the software package ngsRelateV2, which is a major revision of ngsRelateV1. The new software can estimate inbreeding, and estimate relatedness correctly in the presence of inbreeding. The authors demonstrated the accuracy of the method by analyzing some simulated data. It is a valuable paper worth publishing.

I have the following comments for the authors to consider in revision.

1. It is claimed that the 9 IBD configurations cannot be identified and the 9 corresponding IBD coefficients cannot be inferred from biallelic data, such as SNPs.
The reference is
Csűrös, M., 2014. Non-identifiability of identity coefficients at biallelic loci. Theoretical population biology, 92, pp.22-29.
What do you think?

Thank you for pointing out this paper. We observed this problem when working on simulations of complex pedigress with high levels of inbreeding. Csűrös (2014) suggests additional quantities (such as two-out-of-three IBD) which are unambiguous, and we have now included them in out output.(see the wiki documentation for a complete list of summary statistics implemented in ngsRelateV2, https://github.com/angsd/ngsrelate). In addition to these we have added an additional approach for estimating relatedness based on the 2 dimensional site frequency spectrum which is therefore free of assumption of population frequencies. We therefore envision that that software and tool encapsulates a wide variety of existing methods and approaches for estimating relatedness.

2. You missed a relevant paper,

Wang, J., 2007. Triadic IBD coefficients and applications to estimating pairwise relatedness. Genetics Research, 89(3), pp.135-153.

In this paper, a likelihood method was proposed to estimate the 9 condensed IBD coefficients from marker (microsatellites or SNPs) data with possible genotyping errors. The method is implemented in the software Coancestry (the inbreeding model).
Yes we agree completely this paper is very relevant to cite, and we have added the proper citation in the introduction, as reference [8].

3. There are now many published NGS data. Why not analysing a real dataset to demonstrate the new method? Real data are much more complicated than simulated data. But your method and software is for applications to real data, not simulated data.

We agree with the reviewer (See answer 7).

Powered by Editorial Manager® and ProduXion Manager® from Aries Systems Corporation

4. It is unclear to me which algorithm is used to maximize the likelihood for the estimation of the IBD coefficients. How reliable and how fast is the algorithm? Do you need some initial IBD coefficient values to start searching the ML estimates? How do you choose the initial values? Are the results reliant on initial points? These need to be clarified (though not detailed to save space).

We have now clarified the algorithmic details regarding the optimization in a new subsection under “implementation details”.

Reviewer #2: This paper presents a computer program, ngsRelateV2, for the estimation of relatedness parameters from high-throughput sequencing data. The authors use a maximum likelihood based approach to estimate 9 Jacquard coefficients, which is more refined than most empirical studies that use 3 coefficients only. They show the new version of their method can account for inbreeding, and provides better estimates of the Jacquard coefficients in comparison with an earlier version of their method that did not account for inbreeding. This is an interesting and well written paper. I have some major and minor concerns given below.

Major issues

5. The simulations of the authors use several restrictions, which maybe could be relaxed, or should at least be dealt with in more detail. A MAF filter of 0.10 is applied to the founder generation, which is not very realistic, as low MAF variants are in practice more common. What do the authors do when they estimate the Jacquard coefficients by ML? Do they also apply the 0.10 filter to the data prior to estimation? If so, a filter at data generation stage may not be needed. I suggest the authors to repeat the simulations with no and lower (0.05) MAF filter and report the corresponding results, as this will give more information on the performance of their method.

We agree that a MAF filter of 0.05 is probably more commonly used and thus more realistic. We have therefore now performed additional simulations and performed analyses with a 5% allele frequency cutoff (Supplementary figure 1-3). We find that this has no effect on the accuracy of ngsRelate. We have also committed the R script to generate the simulations analyzed in this study to the github (https://github.com/ANGSD/NgsRelate/blob/master/make_simulations.R). The model is based on the existence of population frequencies for diallelic sites, effectively invariant sites will not bring information to the likelihood function.

6. The simulations are carried out in a site-wise manner, by sampling sites independently. Real inheritance is chromosome-wise, with the corresponding LD structure and recombination process. All of this is ignored in the simulations. This implies the authors assume there are as many chromosomes as simulated sites, which is unrealistic. Accounting for all this in the simulations is complicated, but it could be done. The authors should at least address the limitations of their simulations in the discussion section.

We agree that we do not model LD and have now clarified this further in the text (simulation section and implementation details).

7. The authors only work with simulated data, using only 3 very specific scenarios for an FC relationship. They do not give a single empirical data example, which is a serious omission. The analysis of some empirical data could greatly strengthen the paper. There is plenty of empirical data available (e.g. from the 1000 genomes project), and I suggest the authors to analyse all pairs of one population of the 1000G project, and to compare their results with those documented by other researchers (Pemberton et al, 2010).

As suggested by the reviewer, we have analyzed low-coverage NGS data from the 1000 genome project where the relatedness is known. Specifically, we have tested six pairs known to be related in the Luhya in Webuye, Kenya (LWK) population. ngsRelateV2 find estimates overlapping those found with ngsRelateV1. We also show that these individuals display an inbreeding level less than 1%.

Powered by Editorial Manager® and ProduXion Manager® from Aries Systems Corporation

	8. The authors refer to their results as "confidence intervals" (p.2 l.61), but these are boxplots of the values of 100 simulations, which is something very different. Please correct the terminology.<br><br>This is an error that has been fixed.<br><br>Minor issues<br><br>9. Legend figure 2. Acquard coefficients -- Jacquard coefficients.<br>Yes, this has been edited. Thank you for catching it.<br><br>References:<br><br>Pemberton T, Wang C, Li J, Rosenberg N. (2010) Inference of unexpected genetic relatedness among individuals in HapMap Phase III. American Journal of Human Genetics. 87:457-464.<br><br>--<br>Please also take a moment to check our website at https://giga.editorialmanager.com/l.asp?i=45544&l=S42OI455 for any additional comments that were saved as attachments. Please note that as GigaScience has a policy of open peer review, you will be able to see the names of the reviewers.<br><br><br>In compliance with data protection regulations, please contact the publication office if you would like to have your personal information removed from the database.
**Additional Information:**	
**Question**	**Response**
Are you submitting this manuscript to a special series or article collection?	No
**Experimental design and statistics**<br><br><br>Full details of the experimental design and statistical methods used should be given in the Methods section, as detailed in our Minimum Standards Reporting Checklist. Information essential to interpreting the data presented should be made available in the figure legends.<br><br><br>Have you included all the information requested in your manuscript?	Yes
**Resources**<br><br><br>A description of all resources used, including antibodies, cell lines, animals and software tools, with enough information to allow them to be uniquely identified, should be included in the Methods section. Authors are strongly encouraged to cite Research Resource	Yes

Identifiers (RRIDs) for antibodies, model organisms and tools, where possible.<br><br>Have you included the information requested as detailed in our Minimum Standards Reporting Checklist?	
**Availability of data and materials**<br><br>All datasets and code on which the conclusions of the paper rely must be either included in your submission or deposited in publicly available repositories (where available and ethically appropriate), referencing such data using a unique identifier in the references and in the “Availability of Data and Materials” section of your manuscript.<br><br>Have you have met the above requirement as detailed in our Minimum Standards Reporting Checklist?	Yes

Manuscript

Click here to view linked References

Click here to access/download;Manuscript;IBD_GIGASCIENCE.pdf

OXFORD (GIGA)^n SCIENCE

GigaScience, 2018, 1–7

doi: xx.xxxx/xxxx
Manuscript in Preparation
Technical Note

TECHNICAL NOTE

Fast and accurate relatedness estimation from high throughput sequencing data in the presence of inbreeding

Kristian Hanghøj[1,2,*], Ida Moltke[3], Andrea Manica[4] and Thorfinn Sand Korneliussen[1,4,*]

[1]Centre for GeoGenetics, Natural History Museum of Denmark, University of Copenhagen, 1350 Copenhagen K, Denmark and [2]Université de Toulouse, University Paul Sabatier (UPS), Laboratoire AMIS, CNRS UMR 5288, Toulouse, France and [3]Department of Biology, University of Copenhagen, Denmark and [4]Department of Zoology, University of Cambridge, Downing Street, Cambridge CB2 3EJ, UK.

[*]k.hanghoej@snm.ku.dk; tskorneliussen@snm.ku.dk

Abstract

Background: The estimation of relatedness between pairs of possibly inbred individuals from high-throughput sequencing (HTS) data has previously not been possible for samples where we can not obtain reliable genotype calls, as in the case low coverage data.
Results: We introduce ngsRelateV2, a major revision of ngsRelateV1, a program which originally allowed for estimation of relatedness from HTS data among non-inbred individuals only. The new revised version takes into account the possibility of individuals being inbred by estimating the nine condensed Jacquard coefficients along with various other relatedness statistics. The program is threaded and scales linearly with the number of cores allocated to the process.
Conclusion: The program is available as an open source C/C++ program under the GPL license and hosted at https://github.com/ANGSD/ngsRelate. To facilitate easy analysis, the program is able to work directly on the most commonly used container formats for raw sequence (BAM/CRAM) and summary data (VCF/BCF).

Key words: Relatedness estimation; inbreeding; Jacquard coefficients; high throughput sequencing data; genotype likelihood; NGS;threading.

Introduction

Being able to estimate how related two individuals are and whether they are inbred is important in several different fields ranging from conservation genetics to medical genetics. For this purpose, numerous coefficients, like the kinship coefficient and inbreeding coefficients, have been defined and many programs for estimating these coefficients have been proposed.

Notably, the genetic relationship between two individuals can be quantified by the extent to which the two individuals share their alleles identical-by-descent (IBD); i.e. are identical due to recent common ancestry. More specifically, for two diploid individuals, and thus four alleles, there are 15 distinct possible IBD sharing patterns at any given site (detailed identity states). If we ignore the maternal or paternal origin of the alleles, these 15 detailed states can be collapsed into nine condensed states [1] (here denoted $j_1, j_2, \ldots, j_9$), and their corresponding frequency in the genome of two individuals are called the condensed Jacquard coefficients (here denoted $\mathbf{J_1}, \mathbf{J_2}, \ldots, \mathbf{J_9}$). These condensed coefficients provide a comprehensive description of the common ancestry between two individuals, that can be used to infer their familial relationship. Furthermore, many other commonly used coefficients, such as the kinship coeffi-

Compiled on: January 8, 2019.
Draft manuscript prepared by the author.

cient and inbreeding coefficients, can be expressed as linear combinations of the nine condensed Jacquard coefficients.

In the specific case where neither individual is inbred, only three of the condensed Jacquard coefficients can be positive, namely $\mathbf{J_7}$,$\mathbf{J_8}$ and $\mathbf{J_9}$, which are often also denoted $\mathbf{k_2}$,$\mathbf{k_1}$ and $\mathbf{k_0}$, respectively. Numerous approaches, based on either method of moments (e.g. [2]) or maximum-likelihood estimation (e.g. [3]), have been devised to estimate these three quantities assuming that the rest are zero and thus that the individuals are not inbred. This includes commonly used methods like PLINK and KING [2, 4]. Importantly, these methods can lead to wrong estimates and conclusions if applied to inbred individuals because the assumption that only J_7, J_8, and J_9 can be positive is violated. Hence in the presence of inbreeding one needs to estimate all nine coefficients. Several methods for doing this have been proposed [5, 6, 7]. However, very few current tools allow the user to do this and the few that do all require high quality genotype data as input (e.g. [7, 8]). They can therefore not be applied to HTS data of low depth, which is sometimes the only data available. Until recently the same was the case for all the methods for estimating relatedness between non-inbred individual. E.g. both PLINK and KING only work for genotype data. However, recently a few methods that can be applied to low depth sequencing data have been developed [9, 10]. One of these is ngsRelate [10] (hereafter referred to as ngsRelateV1), which works by integrating over every possible genotypic configurations and assigning these a probability given by their genotype likelihood. We here extend this software (hereafter referred to as ngsRelateV2) so it allows the user to infer all nine Jacquard coefficients, and thus allow for inference of relatedness in the presence of inbreeding as well as inbreeding coefficient for both individuals.

Materials & Methods

The underlying statistical framework is similar to that from ngsRelateV1 [10]. Given two individuals, i and j, from the same homogeneous population, we let D_l^i and D_l^j denote the observed HTS data at a biallelic locus l, and G_l^i and G_l^j denote the true, unobserved genotypes at the same locus. Furthermore, we let f_l denote the allele frequency at locus l in the relevant population and X_l denote the unobserved IBD state of the two individuals at locus l. Using this notation we can write the likelihood of the condensed Jacquard coefficients, $\mathbf{J} = (J_1, J_2, J_3, J_4, J_5, J_6, J_7, J_8, J_9)$, for L independent (i.e.unlinked) biallelic loci as:

$$L(J|D^i, D^j, f^A) = \prod_{l=1}^{L} \sum_{m \in \mathbf{J}} P(D_l^i, D_l^j \mid X_l = m, f_l^A) P(X_l = m|J),$$

Notably, here $P(X_l = m \mid J) = J_m$ and $P(D_l^i, D_l^j \mid X_l = m, f_l^A)$ can be rewritten as follows:

$$P(D_l^i, D_l^j \mid X_l = m, f_l^A)$$

$$= \sum_{G_l^i, G_l^j \in \{0,1,2\}^2} P(D_l^i \mid G_l^i) P(D_l^j \mid G_l^j) P(G_l^i, G_l^j \mid f_l^A, X_l = m).$$

where $P(D_l^i|G_l^i)$ and $P(D_l^j|G_l^j)$ denote the per individual genotypes likelihoods for a biallelic locus l, which can be calculated from the sequencing data and $P(G_l^i, G_l^j \mid f_l^A)$ is given from Table 1. We use this likelihood function as a basis for performing maximum likelihood estimation. A number of useful estimates can be calculated directly from J, such as relatedness ($R = J_1 + J_7 + \frac{3}{4}(J_3 + J_5) + \frac{1}{2}J_8$), defined as the proportion of homologous alleles IDB [11], and per individual inbreeding coefficients, F_1 ans F_2 (as in [12]).

We here model the uncertainty of the sequencing data through the genotype likelihoods, but assume knowledge of population frequencies. In the presence of called genotypes (genotypes without uncertainty), our model coincides completely with the approach in [7]. In the absence of inbreeding our model reduces to the work in [10]. We assume that sites are independent, if they are linked our likelihood becomes a composite likelihood that will still have consistent estimates even though it has been shown that it can cause relationships to be overestimated [13, 14].

The novel method assumes that population allele frequencies are obtainable. Although available methods can estimate relatedness without population allele frequencies [15, 9], they do not allow for inbreeding in the two test individuals. We also note that there is an issue of non-identifiability in a context of solely biallelic markers [16], however, ngsRelateV2 estimates a subset of statistics invariant to this that are based on the pairwise genotype distributions. These statistics are calculated directly from the NGS data. See Table 2 for an overview of all the available summary statistics.

Simulations

To simulate data with L sites and N diploid individuals, we first sampled L allele frequencies from a uniform distribution with a minor allele frequency (MAF) filter on 0.05 and 0.1. For each site for each of the N individuals, we sample two alleles using Bernoulli trials with the probability of success equal to the allele frequency for the given site. The outcome of these two trials represent the genotype. Gametes of these individuals are subsequently generated by sampling either of the two alleles from the two haplotypes for every site with equal probability. We assume that each site is independent, thus, linkage disequilibrium (LD) is not modeled. Allosomes are disregarded as well.

From the N founder individuals, we simulate offspring to generate three different pedigrees. From these pedigrees, we have analyzed pairs of individuals with the expected Jacquard coefficients as shown in Table 3.

We then proceed by calculating genotype likelihoods by assuming different sequencing depths $d = \{1X, 2X, 4X, 8X, 16X\}$, error rate $e = 0.001$ and number of sites $s = \{10K, 30K, 50K\}$ for the individuals of interest. The per-site-per-individual sequencing depth is given by sampling the depth from a Poisson distribution with parameter d and using the binomial density distribution with e. This approach is similar to the previous approach in [10] which does not model the spatial properties of true recombination and LD.

Results

To test the performance of ngsRelateV2, we use three simulated scenarios (see Simulations section) and compare it to ngsRelateV1 [10]. For every scenario, we generate 100 independent simulations for every combination of sequencing effort and number of segregating sites. In the first scenario, we compare two outbred cousins (fig.1). As expected, both versions of ngsRelate find not only the correct level of relatedness, but also the correct estimates of the three relevant Jacquard coefficients (J_7, J_8, J_9). The second scenario also includes two cousins, but this time we have introduced inbreeding in one of the individu-

Table 1. Probabilities for various allelic states, given modes of IDB from Table 1 in [7]. Triallelic sites are disregarded.

Allelic State	J_1	J_2	J_3	J_4	J_5	J_6	J_7	J_8	J_9
$A_iA_iA_iA_i$	p_i	p_i^2	p_i^2	p_i^3	p_i^2	p_i^3	p_i^2	p_i^3	p_i^4
$A_iA_iA_jA_j$	0	p_ip_j	0	p_ip_j	0	$p_i^2p_j$	0	0	$p_i^2p_j^2$
$A_iA_iA_iA_j$	0	0	p_ip_j	$2p_i^2p_j$	0	0	0	$p_i^2p_j$	$2p_i^3p_j$
$A_iA_jA_iA_i$	0	0	0	0	p_ip_j	$2p_i^2p_j$	0	$p_i^2p_j$	$2p_i^3p_j$
$A_iA_jA_iA_j$	0	0	0	0	0	0	$2p_ip_j$	p_ip_j	$4p_i^2p_j^2$

Table 2. Various relatedness statistics estimated from ngsRelateV2 and which summary statistics they are based on.

Statistics	Formula	summary statistic	Reference
r_{ab}	$(J_1 + J_7 + 0.75 * (J_3 + J_5) + .5 * J_8)$	IBD	[11]
F_a	$(J_1 + J_2 + J_3 + J_4)$	IBD	[17]
F_b	$(J_1 + J_2 + J_5 + J_6)$	IBD	[17]
θ	$J_1 + 0.5 * (J_3 + J_5 + J_7) + 0.25 * J_8$	IBD	[17]
F_{12}	$J_1 + 0.5 * J_3$	IBD	[11]
F_{21}	$J_1 + 0.5 * J_5$	IBD	[11]
Fraternity	$J_2 + J_7$	IBD	[18]
Identity	J_1	IBD	[18]
Zygosity	$J_1 + J_2 + J7$	IBD	[18]
2-3-IBD	$J_1 + J_2 + J_3 + J_5 + J_7 + 0.5 * (J_4 + J_6 + J_8)$	IBD	[16]
F_{diff}	$0.5 * (J_4 - J_6)$	IBD	[16]
R_0	$(C + G)/E$	IBS	[15]
R_1	$E/(B + D + H + F + C + G)$	IBS	[15]
King	$(E - 2(C + G))/(B + D + H + F + 2 * E)$	IBS	[15]

Table 3. Expected Jacquard coefficients, relatedness and inbreeding coefficients for three simulated scenarios.

	J_1	J_2	J_3	J_4	J_5	J_6	J_7	J_8	J_9	R	F_1	F_2
scenario$_1$	0	0	0	0	0	0	0	0.25	0.75	0.13	0	0
scenario$_2$	0	0	0	0	0.06	0.19	0	0.38	0.38	0.23	0	0.25
scenario$_3$	0.02	0.02	0.09	0.12	0.06	0.06	0.06	0.38	0.22	0.38	0.25	0.13

als. The parents of the inbred individual are related corresponding to a parent-child relation. In this scenario, even at low sequencing effort and only 10k sites, ngsRelateV2 correctly estimates the coefficients of relatedness and inbreeding; however, the estimates of the nine Jacquard coefficients are somewhat noisy, and at least 50k segregating sites are needed to increase the accuracy (fig.2). The final scenario, being the most complex, includes the inbred individual from scenario two and another inbred cousin with its parents being related corresponding to a grandparent-grandchild relation. Interestingly, with such a complex pedigree, ngsRelateV2 still manages to recover the exact estimates for relatedness and individual inbreeding coefficients, even with only 10k segregating sites and a low sequencing depth (fig.3). Similarly to the results from scenario two, confident estimates of the nine Jacquard coefficients required increasing the number of informative sites and/or the sequencing effort. We also applied ngsRelateV2 to these three scenarios using a MAF cutoff on 0.05 (Supplementary Figure 1-3). We find that ngsRelateV2 recovers comparable accuracy with a MAF filter on 0.05.

We also applied ngsRelateV2 to real HTS data and compared the estimates to those obtained with ngsRelateV1. We used six pairwise related genomes, sequenced to low coverage (approximately 4X), from the LWK population generated as part of the 1000 Genomes Project [19]. We calculated genotype likelihoods of the related individuals, using ANGSD [20], at genomic sites with MAF in the LWK population on 0.05, summing up to 4.6m segregating sites. We not only show that ngsRelateV2 obtains comparable relatedness estimates to those obtained by ngsRelateV1, with this novel software, we also show that all the tested individuals show an inbreeding coefficient below 1% (Supplementary Figure 4).

In extremely complicated pedigrees with symmetric inbreeding, such as multiple generations of full sibling mating, we find multiple global maxima where several combinations of the nine Jacquard coefficients, including the expected coefficients, are equally likely. Albeit observing such identifiability challenges, we, importantly, still find accurate relatedness estimates and individual inbreeding coefficients by summing the relevant Jacquard coefficients.

For every pair of individuals, ngsRelateV2 generates and outputs estimates of the nine Jacquard coefficients, the relatedness, the individual inbreeding coefficients as described above but also other combinations of the nine Jacquard coefficient: the kinship coefficient, fraternity, and the three summary statistics inbred relatedness, identity, and zygosity, suggested by Ackerman and colleagues [18]. It also produces the KING statistic [21] based on the two dimensional site frequency spectrum of pairs of individuals following the methodology in [15]. The latter statistics do not require population allele frequencies. Thanks to threading, ngsRelateV2 scales well to large numbers of pairwise comparisons of individuals.

Conclusion

The tool presented in this technical note allows researchers to perform relatedness analysis for inbred individuals in a statistical framework that is especially suited for low coverage sequence data. The results show that the method performs well for estimating all nine coefficients, at least when the underlying pedigrees are not extremely complex. And even when the underlying pedigree is very complex, compound summaries of the output, like relatedness and inbreeding coefficients, will still be correct. The implementation is a fast multi threaded C++ program that can be directly applied to

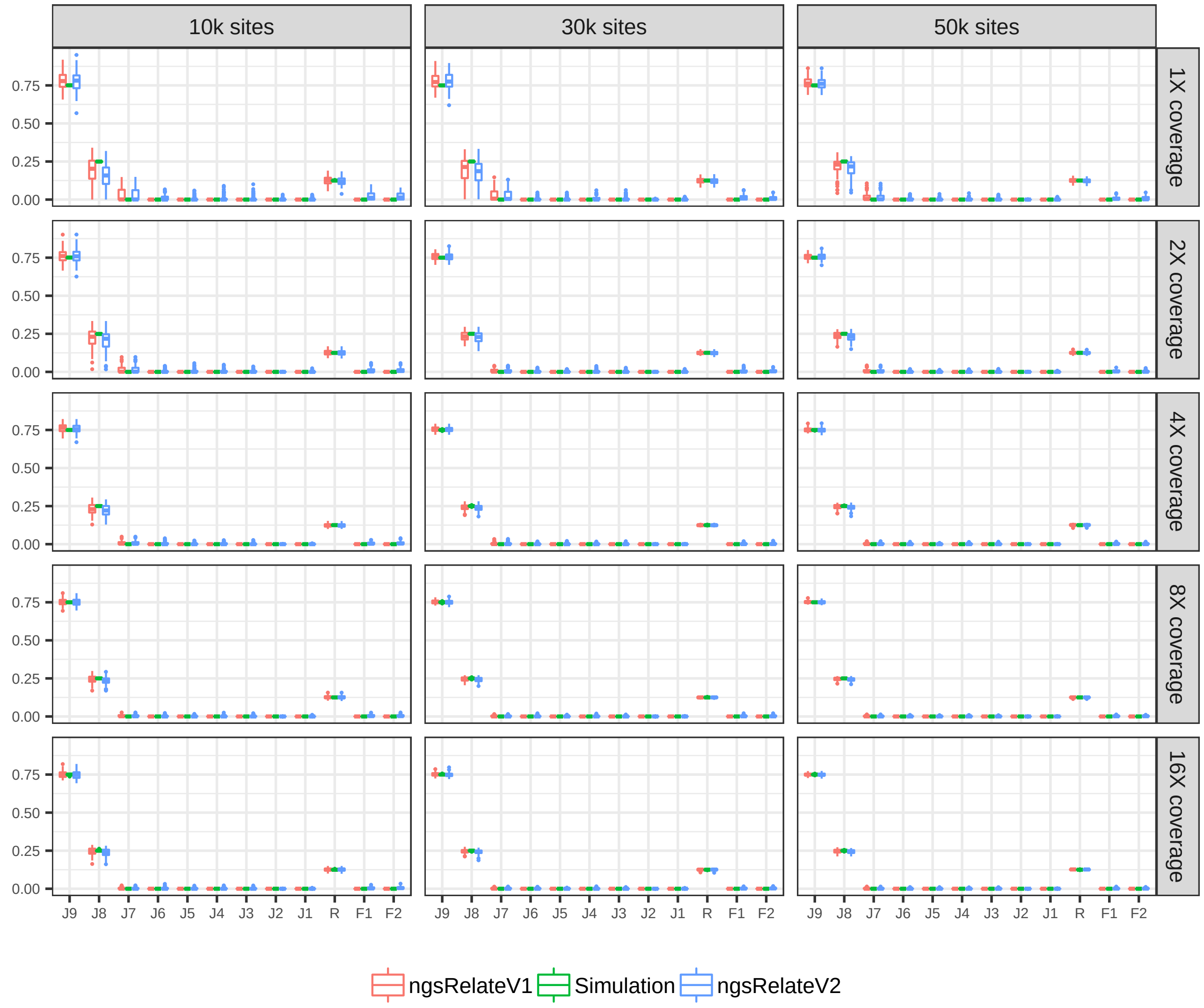

Figure 1. 100 independent simulations of two outbred cousins across variable sequencing depth and informative sites with a minor allele frequence cutoff on 10%. J_9 to J_1 refer to the nine Jacquard coefficients, R is the relatedness, finally, F_1 and F_2 refer to the individual inbreeding coefficients. Simulation (green) are the true values that we compare ngsRelateV1 (red) and the new program ngsRelateV2 (blue) against.

the most commonly used data files used for high throughput sequencing data.

Implementation Details

The program is implemented in a fast multithreaded c++ program and takes as input either genotype likelihood files and frequencies or bcf/vcf files as produced from standard tools such as GATK[22] or SAMtools [23]. We also include an R implementation that we used for simulating data. Of note, the simulations generated in this study do not accounted for LD. In case of LD between genetic variants, the likelihood function becomes a composite likelihood function. The maximum likelihood estimate of such a function is consistent to that found with a likelihood function of independent sites [24].

The optimization follows the approach described in [10]. The optimization is an accelerated expectation maximization (EM) following the squared iterative approach in **S3** in [25] and is initialized with a random start point within the parameter space. The borders of the parameter space are manually examined after convergence. Since the EM algorithm is only guaranteed to find a local optimum, it is recommended to rerun with multiple different seeds though we note that we did not find an issue with multiple local optima in our examples.

Availability of source code and requirements

- Project name: ngsRelateV2
- Project home page: http://github.com/ANGSD/ngsRelate
- Operating system(s): Platform independent
- Programming language: C++
- Other requirements: htslib (only for parsing VCF/BCF files)
- License: GNU GPL (version 3)
- RRID: SCR_016588

Declarations

List of abbreviations

IBD: Identity-by-descent. HTS: High-throughput sequencing.

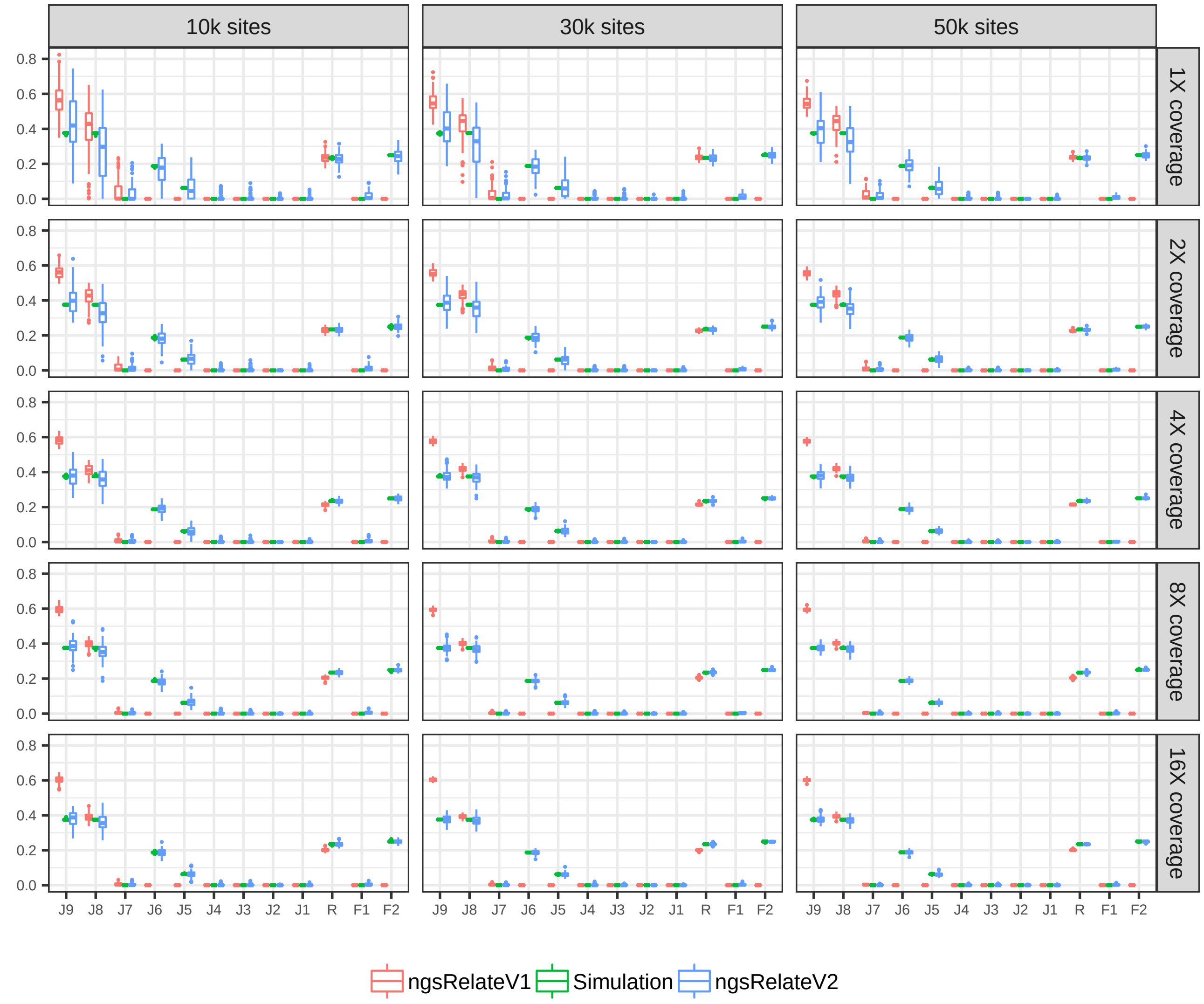

Figure 2. 100 independent simulations of two cousins, with one individual being inbred, across variable sequencing depth and segregating sites with a minor allele frequence cutoff on 10%. J_9 to J_1 refer to the nine Jacquard coefficients, R is the relatedness, finally, F_1 and F_2 refer to the individual inbreeding coefficients. Simulation (green) are the true values that we compare ngsRelateV1 (red) and the new program ngsRelateV2 (blue) against.

Consent for publication

Not applicable.

Competing Interests

None.

Funding

KH is funded by the Danish National Research Foundation (DNRF94) and the Initiative d'Excellence Chaires d'attractivité, Université de Toulouse (OURASI); TSK by a grant from the Carlsberg Foundation (CF16-0913); IM by Independent Research Fund Denmark (DFF – 4090-00244); AM by an ERC Consolidator Grant LocalAdaptation 647787.

Authors' Contributions

TSK devised the model. KH implemented and ran all analyses. IM and AM devised test scenarios and improved early versions of the method. All authors wrote the article.

Acknowledgements

The program was first prototyped by Philip Alstrup Andersen, a Master student under the supervision of TSK and IM.

References

1. Weir BS, Anderson AD, Hepler AB. Genetic relatedness analysis: modern data and new challenges. Nat Rev Genet 2006 Oct;7(10):771–780.
2. Purcell S, Neale B, Todd-Brown K, Thomas L, Ferreira MA, Bender D, et al. PLINK: a tool set for whole-genome association and population-based linkage analyses. Am J Hum Genet 2007 Sep;81(3):559–575.
3. Thompson EA. The estimation of pairwise relationships. Ann Hum Genet 1975 Oct;39(2):173–188.
4. Manichaikul A, Mychaleckyj JC, Rich SS, Daly K, Sale M, Chen WM. Robust relationship inference in genome-wide association studies. Bioinformatics 2010 Nov;26(22):2867–

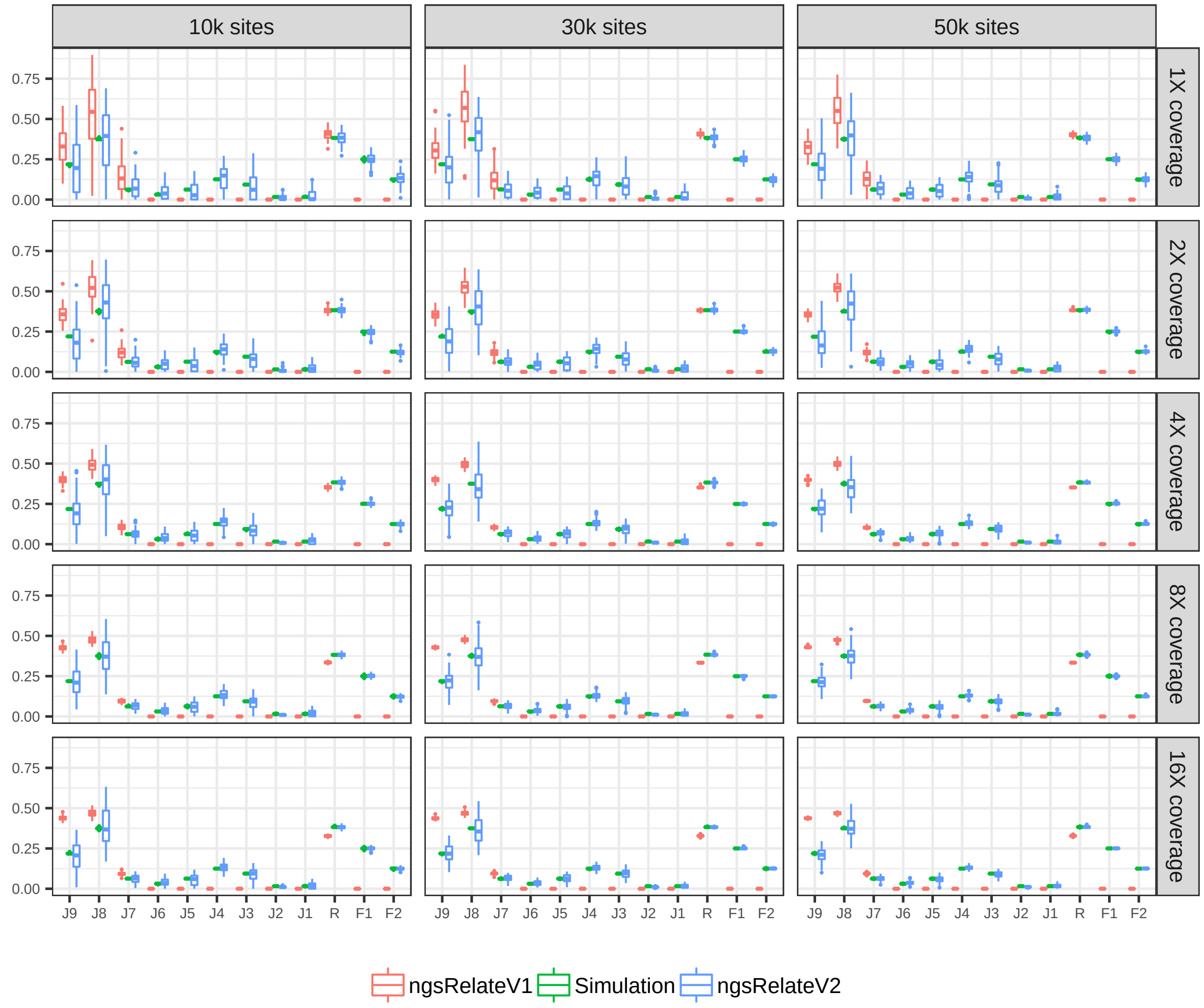

Figure 3. 100 independent simulations of two cousins, both being inbred, across variable sequencing depth and segregating sites with a minor allele frequence cutoff on 10%. J_9 to J_1 refer to the nine Jacquard coefficients, R is the relatedness, finally, F_1 and F_2 refer to the individual inbreeding coefficients. Simulation (green) are the true values that we compare ngsRelateV1 (red) and the new program ngsRelateV2 (blue) against.

2873.

5. Ritland K. Estimators for pairwise relatedness and individual inbreeding coefficients. Genetical Research 1996;67(2):175–185.
6. Milligan BG. Maximum-likelihood estimation of relatedness. Genetics 2003 Mar;163(3):1153–1167.
7. Anderson AD, Weir BS. A maximum-likelihood method for the estimation of pairwise relatedness in structured populations. Genetics 2007 May;176(1):421–440.
8. Wang J. COANCESTRY: a program for simulating, estimating and analysing relatedness and inbreeding coefficients. Mol Ecol Resour 2011 Jan;11(1):141–145.
9. Kuhn JMM, Jakobsson M, Gunther T. Estimating genetic kin relationships in prehistoric populations. PLoS ONE 2018;13(4):e0195491.
10. Korneliussen TS, Moltke I. NgsRelate: a software tool for estimating pairwise relatedness from next-generation sequencing data. Bioinformatics 2015 Dec;31(24):4009–4011.
11. Hedrick PW, Lacy RC. Measuring Relatedness between Inbred Individuals. Journal of Heredity 2015;106(1):20–25. http://dx.doi.org/10.1093/jhered/esu072.
12. Vieira FG, Fumagalli M, Albrechtsen A, Nielsen R. Estimating inbreeding coefficients from NGS data: Impact on genotype calling and allele frequency estimation. Genome Res 2013 Nov;23(11):1852–1861.
13. Ko A, Nielsen R. Composite likelihood method for inferring local pedigrees. PLoS Genet 2017 Aug;13(8):e1006963.
14. Sun M, Jobling MA, Taliun D, Pramstaller PP, Egeland T, Sheehan NA. On the use of dense SNP marker data for the identification of distant relative pairs. Theor Popul Biol 2016 Feb;107:14–25.
15. Waples RK, Albrechtsen A, Moltke I. Allele frequency-free inference of close familial relationships from genotypes or low depth sequencing data. bioRxiv 2018;https://www.biorxiv.org/content/early/2018/08/31/260497.
16. Cs?ros M. Non-identifiability of identity coefficients at biallelic loci. Theor Popul Biol 2014 Mar;92:22–29.
17. Jacquard A. The genetic structure of populations, vol. 5. Springer Science & Business Media; 2012.
18. Ackerman MS, Johri P, Spitze K, Xu S, Doak TG, Young K, et al. Estimating Seven Coefficients of Pairwise Relatedness Using Population-Genomic Data. Genetics 2017 05;206(1):105–118.
19. Consortium GP, et al. An integrated map of genetic variation from 1,092 human genomes. Nature 2012;491(7422):56.

20. Korneliussen TS, Albrechtsen A, Nielsen R. ANGSD: Analysis of Next Generation Sequencing Data. BMC Bioinformatics 2014 Nov;15(1):356. https://doi.org/10.1186/s12859-014-0356-4.
21. Manichaikul A, Mychaleckyj JC, Rich SS, Daly K, Sale M, Chen WM. Robust relationship inference in genome-wide association studies. Bioinformatics 2010 Nov;26(22):2867–2873.
22. McKenna A, Hanna M, Banks E, Sivachenko A, Cibulskis K, Kernytsky A, et al. The Genome Analysis Toolkit: a MapReduce framework for analyzing next-generation DNA sequencing data. Genome Res 2010 Sep;20(9):1297–1303.
23. Li H, Handsaker B, Wysoker A, Fennell T, Ruan J, Homer N, et al. The Sequence Alignment/Map format and SAMtools. Bioinformatics 2009 Aug;25(16):2078–2079.
24. Lindsay BG. Composite likelihood methods. Contemporary mathematics 1988;80(1):221–239.
25. VARADHAN R, ROLAND C. Simple and Globally Convergent Methods for Accelerating the Convergence of Any EM Algorithm. Scandinavian Journal of Statistics;35(2):335–353. https://onlinelibrary.wiley.com/doi/abs/10.1111/j.1467-9469.2007.00585.x.

Supplementary Material

Click here to access/download
Supplementary Material
suppl.pdf
