# Supplementary material for: Fast and accurate relatedness estimation from high-throughput sequencing data in the presence of inbreeding
Source: Gigascience. 2019 Apr 30;8(5):giz034. doi: 10.1093/gigascience/giz034 (PMC6488770; doi:10.1093/gigascience/giz034)
Supplement: GIGA-D-18-00338_Revision-2.pdf [file giz034_giga-d-18-00338_revision-2.pdf]

GigaScience

Fast and accurate relatedness estimation from high throughput sequencing data in the presence of inbreeding

--Manuscript Draft--

**Manuscript Number:**	GIGA-D-18-00338R2
**Full Title:**	Fast and accurate relatedness estimation from high throughput sequencing data in the presence of inbreeding
**Article Type:**	Technical Note
**Funding Information:**	Carlsbergfondet (DK) (CF16-0913) — Dr Thorfinn Sand Korneliussen<br>Danmarks Grundforskningsfond (DK) (DNRF94) — Mr Kristian Hanghoej<br>initiative d'Excellence Chaires d'attractivité (OURASI) — Mr Kristian Hanghoej<br>Ydun (NA) — Dr Ida Moltke<br>ERC consolidator grant (LocalAdaptation 647787) — Dr Andrea Manica<br>Danmarks Grundforskningsfond (DK) (DNRF 0094) — Dr Thorfinn Sand Korneliussen<br>Lundbeckfonden (R302-2018-2155) — Dr Thorfinn Sand Korneliussen
**Abstract:**	Background: The estimation of relatedness between pairs of possibly inbred individuals from high-throughput sequencing (HTS) data has previously not been possible for samples where we can not obtain reliable genotype calls, as in the case of low coverage data.<br>Results: We introduce ngsRelateV2, a major revision of ngsRelateV1, a program which originally allowed for estimation of relatedness from HTS data among non-inbred individuals only. The new revised version takes into account the possibility of individuals being inbred by estimating the nine condensed Jacquard coefficients along with various other relatedness statistics. The program is threaded and scales linearly with the number of cores allocated to the process.<br>Conclusion: The program is available as an open source \texttt{C/C++} program under the GPL license and hosted at https://github.com/ANGSD/ngsRelate. To facilitate easy analysis, the program is able to work directly on the most commonly used container formats for raw sequence (BAM/CRAM) and summary data (VCF/BCF).
**Corresponding Author:**	Thorfinn Sand Korneliussen, Ph.D<br>Natural History Museum of Denmark<br>Copenhagen, DENMARK
**Corresponding Author Secondary Information:**	
**Corresponding Author's Institution:**	Natural History Museum of Denmark
**Corresponding Author's Secondary Institution:**	
**First Author:**	Kristian Hanghoej, msc
**First Author Secondary Information:**	
**Order of Authors:**	Kristian Hanghoej, msc<br>Ida Moltke, PhD<br>Andrea Manica, PhD<br>Thorfinn Sand Korneliussen, Ph.D<br>Philip Alstrup Andersen
**Order of Authors Secondary Information:**	

Powered by Editorial Manager® and ProduXion Manager® from Aries Systems Corporation

Response to Reviewers:	We have fixed all comments from reviewer, which we found very useful and helpful.<br><br>Additionally:<br>We have further extended ngsRelateV2 to take plink files as input.<br>We have also added a figure to show that the threading works as expected along with runtime estimates. (Figure 5) and a table (Table 4) that also shows memory usage for different number of threads allocated to the program<br>Implementation wise we have added the possibility of doing bootstrap replicates along with performing multiple optimizations on the fly to circumvent the issue of local optima<br>Added missing funding bodies.<br>Added the reviewers comments in the acknowledgement<br>Added an additional coauthor. The coauthor that has been added was working on this as part of his MSc project.<br>We have added a citation to the associated gigadb entry<br><br>Reviewer reports:<br>Reviewer #2: The authors have updated their manuscript, taking most of the suggestions of the reviewers satisfactorily into account. I have some additional comments, mostly minor, that may be of help for the authors to further improve their manuscript.<br><br>Abstract: "In the case low coverage" --> "in the case of low coverage"<br>DONE<br>Pg. 2, l. 4. The authors may wish to add "respectively, and known as Cotterman's coefficients (Cotterman, 1941)."<br>DONE<br>Pg. 2, l. 28. "as well as inbreeding coefficient" --> "as well as inference of the inbreeding coefficients"<br>DONE<br>Pg. 2, l. 6, 2nd col. "population frequencies" --> "population allele frequencies"<br>DONE<br>Pg. 2, l. 30, 2nd col. "Bernoulli trials" --> "independent Bernoulli trails"<br>DONE<br>Pg. 2, l. 30, 2nd col. "given site." The authors may wish to add: "given site, implying the data is generated under the assumption of Hardy-Weinberg equilibrium."<br>DONE<br>Pg. 3, l. 8: "estimated from" --> "estimated by"<br>DONE<br>Pg. 3, l. 18: J7; the "7" should be a subscript.<br>DONE<br>Pg. 3, l. 33: It is not clear what is meant with "confident estimates". Improved estimates?<br>We have changed it to “accurate estimates”<br><br>Pg. 3, l. 60: Supplementary figure 4. This is an important application showing that the proposed estimation procedure seems to work well in practice. I suggest the figure not to be supplementary, but part of the main manuscript. The documented relationships of the six panels should be clearly indicated, for instance by putting (PO), (FS) or whatever corresponds in the banner of each panel behind the identifiers of each pair. Documented relationships could be mentioned in the text or the caption of the figure as well.<br><br><br>We have moved supplementary figure 4 to the main manuscript and added the pairwise relationships to the figure.<br><br>The authors have apparently been a bit careless in their update of the bibliography. Please address the following issues:<br><br>16: the author name is misspelled.<br>DONE<br>17. This book is not from 2015, but from 1974. Please check out the correct reference.<br>DONE<br>19. The author, the 1000G Consortium, is not correctly spelled out.

Powered by Editorial Manager® and ProduXion Manager® from Aries Systems Corporation

	DONE<br>25. Surnames not fully in uppercase.<br>DONE
**Additional Information:**	
**Question**	**Response**
Are you submitting this manuscript to a special series or article collection?	No
**Experimental design and statistics**<br><br>Full details of the experimental design and statistical methods used should be given in the Methods section, as detailed in our Minimum Standards Reporting Checklist. Information essential to interpreting the data presented should be made available in the figure legends.<br><br>Have you included all the information requested in your manuscript?	Yes
**Resources**<br><br>A description of all resources used, including antibodies, cell lines, animals and software tools, with enough information to allow them to be uniquely identified, should be included in the Methods section. Authors are strongly encouraged to cite Research Resource Identifiers (RRIDs) for antibodies, model organisms and tools, where possible.<br><br>Have you included the information requested as detailed in our Minimum Standards Reporting Checklist?	Yes
**Availability of data and materials**<br><br>All datasets and code on which the conclusions of the paper rely must be either included in your submission or deposited in publicly available repositories (where available and ethically appropriate), referencing such data using a unique identifier in the references and in	Yes

the “Availability of Data and Materials” section of your manuscript.<br><br>Have you have met the above requirement as detailed in our Minimum Standards Reporting Checklist?	

Powered by Editorial Manager® and ProduXion Manager® from Aries Systems Corporation

Manuscript
Click here to view linked References
Click here to access/download;Manuscript;IBD_GIGASCIENCE-7.pdf

OXFORD (GIGA)^n SCIENCE

GigaScience, 2018, 1–9

doi: xx.xxxx/xxxx
Manuscript in Preparation
Technical Note

TECHNICAL NOTE

Fast and accurate relatedness estimation from high throughput sequencing data in the presence of inbreeding

Kristian Hanghøj[1,2,*], Ida Moltke[3], Philip Alstrup Andersen[3], Andrea Manica[4] and Thorfinn Sand Korneliussen[1,4,*]

[1]Centre for GeoGenetics, Natural History Museum of Denmark, University of Copenhagen, 1350 Copenhagen K, Denmark and [2]Université de Toulouse, University Paul Sabatier (UPS), Laboratoire AMIS, CNRS UMR 5288, Toulouse, France and [3]Department of Biology, University of Copenhagen, Denmark and [4]Department of Zoology, University of Cambridge, Downing Street, Cambridge CB2 3EJ, UK.

[*]k.hanghoej@snm.ku.dk; tskorneliussen@snm.ku.dk

Abstract

Background: The estimation of relatedness between pairs of possibly inbred individuals from high-throughput sequencing (HTS) data has previously not been possible for samples where we can not obtain reliable genotype calls, as in the case of low coverage data.
Results: We introduce ngsRelateV2, a major revision of ngsRelateV1, a program which originally allowed for estimation of relatedness from HTS data among non-inbred individuals only. The new revised version takes into account the possibility of individuals being inbred by estimating the nine condensed Jacquard coefficients along with various other relatedness statistics. The program is threaded and scales linearly with the number of cores allocated to the process.
Conclusion: The program is available as an open source C/C++ program under the GPL license and hosted at https://github.com/ANGSD/ngsRelate. To facilitate easy analysis, the program is able to work directly on the most commonly used container formats for raw sequence (BAM/CRAM) and summary data (VCF/BCF).

Key words: Relatedness estimation; inbreeding; Jacquard coefficients; high throughput sequencing data; genotype likelihood; NGS;threading.

Introduction

Being able to estimate how related two individuals are and whether they are inbred is important in several different fields ranging from conservation genetics to medical genetics. For this purpose, numerous coefficients, like the kinship coefficient and inbreeding coefficients, have been defined and many programs for estimating these coefficients have been proposed.

Notably, the genetic relationship between two individuals can be quantified by the extent to which the two individuals share their alleles identical-by-descent (IBD); i.e. are identical due to recent common ancestry. More specifically, for two diploid individuals, and thus four alleles, there are 15 distinct possible IBD sharing patterns at any given site (detailed identity states). If we ignore the maternal or paternal origin of the alleles, these 15 detailed states can be collapsed into nine condensed states [1] (here denoted $j_1, j_2, \ldots, j_9$), and their corresponding frequency in the genome of two individuals are called the condensed Jacquard coefficients (here denoted $\mathbf{J_1}, \mathbf{J_2}, \ldots, \mathbf{J_9}$). These condensed coefficients provide a comprehensive description of the common ancestry between two individuals, that can be used to infer their familial relationship. Furthermore, many other commonly used coefficients, such as the kinship coeffi-

Compiled on: March 8, 2019.
Draft manuscript prepared by the author.

cient and inbreeding coefficients, can be expressed as linear combinations of the nine condensed Jacquard coefficients.

In the specific case where neither individual is inbred, only three of the condensed Jacquard coefficients can be positive, namely $\mathbf{J_7}$,$\mathbf{J_8}$ and $\mathbf{J_9}$, which are often also denoted $\mathbf{k_2}$,$\mathbf{k_1}$ and $\mathbf{k_0}$, respectively, and known as Cotterman's coefficients [2]. Numerous approaches, based on either method of moments (e.g. [3]) or maximum-likelihood estimation (e.g. [4]), have been devised to estimate these three quantities assuming that the rest are zero and thus that the individuals are not inbred. This includes commonly used methods like PLINK and KING [3, 5]. Importantly, these methods can lead to wrong estimates and conclusions if applied to inbred individuals because the assumption that only J_7, J_8, and J_9 can be positive is violated. Hence in the presence of inbreeding one needs to estimate all nine coefficients. Several methods for doing this have been proposed [6, 7, 8]. However, very few current tools allow the user to do this and the few that do all require high quality genotype data as input (e.g. [8, 9]). They can therefore not be applied to HTS data of low depth, which is sometimes the only data available. Until recently the same was the case for all the methods for estimating relatedness between non-inbred individual. E.g. both PLINK and KING only work for genotype data. However, recently a few methods that can be applied to low depth sequencing data have been developed [10, 11]. One of these is ngsRelate [11] (hereafter referred to as ngsRelateV1), which works by integrating over every possible genotypic configurations and assigning these a probability given by their genotype likelihood. We here extend this software (hereafter referred to as ngsRelateV2) so it allows the user to infer all nine Jacquard coefficients, and thus allow for inference of relatedness in the presence of inbreeding as well as inference of the inbreeding coefficients for both individuals.

Materials & Methods

The underlying statistical framework is similar to that from ngsRelateV1 [11]. Given two individuals, i and j, from the same homogeneous population, we let D^i_l and D^j_l denote the observed HTS data at a biallelic locus l, and G^i_l and G^j_l denote the true, unobserved genotypes at the same locus. Furthermore, we let f_l denote the allele frequency at locus l in the relevant population and X_l denote the unobserved IBD state of the two individuals at locus l. Using this notation we can write the likelihood of the condensed Jacquard coefficients, $\mathbf{J} = (J_1, J_2, J_3, J_4, J_5, J_6, J_7, J_8, J_9)$, for L independent (i.e.unlinked) biallelic loci as:

$$L(J|D^i, D^j, f^A) = \prod_{l=1}^{L} \sum_{m \in \mathbf{J}} P(D^i_l, D^j_l \mid X_l = m, f^A_l) P(X_l = m|J),$$

Notably, here $P(X_l = m \mid J) = J_m$ and $P(D^i_l, D^j_l \mid X_l = m, f^A_l)$ can be rewritten as follows:

$$P(D^i_l, D^j_l \mid X_l = m, f^A_l)$$

$$= \sum_{G^i_l, G^j_l \in \{0,1,2\}^2} P(D^i_l \mid G^i_l) P(D^j_l \mid G^j_l) P(G^i_l, G^j_l \mid f^A_l, X_l = m).$$

where $P(D^i_l|G^i_l)$ and $P(D^j_l|G^j_l)$ denote the per individual genotypes likelihoods for a biallelic locus l, which can be calculated from the sequencing data and $P(G^i_l, G^j_l \mid f^A_l)$ is given from Table 1. We use this likelihood function as a basis for performing maximum likelihood estimation. A number of useful estimates can be calculated directly from J, such as relatedness ($R = J_1 + J_7 + \frac{3}{4}(J_3 + J_5) + \frac{1}{2} J_8$), defined as the proportion of homologous alleles IDB [12], and per individual inbreeding coefficients, F_1 ans F_2 (as in [13]).

We here model the uncertainty of the sequencing data through the genotype likelihoods, but assume knowledge of population allele frequencies. In the presence of called genotypes (genotypes without uncertainty), our model coincides completely with the approach in [8]. In the absence of inbreeding our model reduces to the work in [11]. We assume that sites are independent, if they are linked our likelihood becomes a composite likelihood that will still have consistent estimates even though it has been shown that it can cause relationships to be overestimated [14, 15].

This novel method assumes that populations allele frequencies are obtainable, and we note that it has been shown in [16] that working in a context of solely diallelic markers the estimation of the the nine condensed Jaquard coefficients can display an issue of non-identifiability. This will have an impact for some of summary statistics that are defined as linear combinations of these coefficients, with the estimators that are invariant being R, F_a,F_b, θ, $2-3-IBD$, F_{diff}. Finally ngsRelateV2 also computes three summary statistics (Table 2, IBS) based on the 2D-SFS [17], but note that summary statistics based on the 2D-sfs do not require known population allele frequencies, they assume the individuals to be non-inbred. The 2D-SFS obtained in ngsRelateV2 follows the methodology from [18] that is based on genotype likelihoods and does therefore not require called genotypes.

In addition to the raw statistics we have also developed a bootstrapping approach that can be used to recover confidence intervals of all the summary statistics shown in Table 2.

Simulations

To simulate data with L sites and N diploid individuals, we first sampled L allele frequencies from a uniform distribution with a minor allele frequency (MAF) filter on 0.05 and 0.1. For each site for each of the N individuals, we sample two alleles using indepedent Bernoulli trials with the probability of success equal to the allele frequency for the given site, implying the data is generated under the assumption of Hardy-Weinberg equilibrium. The outcome of these two trials represent the genotype. Gametes of these individuals are subsequently generated by sampling either of the two alleles from the two haplotypes for every site with equal probability. We assume that each site is independent, thus, linkage disequilibrium (LD) is not modeled. Allosomes are disregarded as well.

From the N founder individuals, we simulate offspring to generate three different pedigrees. From these pedigrees, we have analyzed pairs of individuals with the expected Jacquard coefficients as shown in Table 3.

We then proceed by calculating genotype likelihoods by assuming different sequencing depths $d = \{1X, 2X, 4X, 8X, 16X\}$, error rate $e = 0.001$ and number of sites $s = \{10K, 30K, 50K\}$ for the individuals of interest. The per-site-per-individual sequencing depth is given by sampling the depth from a Poisson distribution with parameter d and using the binomial density distribution with e. This approach is similar to the previous approach in [11] which does not model the spatial properties of true recombination and LD.

Table 1. Probabilities for various allelic states, given modes of IDB from Table 1 in [8]. Triallelic sites are disregarded.

Allelic State	J_1	J_2	J_3	J_4	J_5	J_6	J_7	J_8	J_9
$A_iA_iA_iA_i$	p_i	p_i^2	p_i^2	p_i^3	p_i^2	p_i^3	p_i^2	p_i^3	p_i^4
$A_iA_iA_jA_j$	0	p_ip_j	0	p_ip_j	0	$p_i^2p_j$	0	0	$p_i^2p_j^2$
$A_iA_iA_iA_j$	0	0	p_ip_j	$2p_i^2p_j$	0	0	0	$p_i^2p_j$	$2p_i^3p_j$
$A_iA_jA_iA_i$	0	0	0	0	p_ip_j	$2p_i^2p_j$	0	$p_i^2p_j$	$2p_i^3p_j$
$A_iA_jA_iA_j$	0	0	0	0	0	0	$2p_ip_j$	p_ip_j	$4p_i^2p_j^2$

Table 2. Various relatedness statistics estimated by ngsRelateV2 and which summary statistics they are based on.

Statistics	Formula	summary statistic	Reference
r_{ab}	$(J_1 + J_7 + 0.75 * (J_3 + J_5) + .5 * J_8)$	IBD	[12]
F_a	$(J_1 + J_2 + J_3 + J_4)$	IBD	[19]
F_b	$(J_1 + J_2 + J_5 + J_6)$	IBD	[19]
θ	$J_1 + 0.5 * (J_3 + J_5 + J_7) + 0.25 * J_8$	IBD	[19]
F_{12}	$J_1 + 0.5 * J_3$	IBD	[12]
F_{21}	$J_1 + 0.5 * J_5$	IBD	[12]
Fraternity	$J_2 + J_7$	IBD	[20]
Identity	J_1	IBD	[20]
Zygosity	$J_1 + J_2 + J_7$	IBD	[20]
2-3-IBD	$J_1 + J_2 + J_3 + J_5 + J_7 + 0.5 * (J_4 + J_6 + J_8)$	IBD	[16]
F_{diff}	$0.5 * (J_4 - J_6)$	IBD	[16]
R_0	$(C + G)/E$	IBS	[17]
R_1	$E/(B + D + H + F + C + G)$	IBS	[17]
King	$(E - 2(C + G))/(B + D + H + F + 2 * E)$	IBS	[17]

Table 3. Expected Jacquard coefficients, relatedness and inbreeding coefficients for three simulated scenarios.

	J_1	J_2	J_3	J_4	J_5	J_6	J_7	J_8	J_9	R	F_1	F_2
$scenario_1$	0	0	0	0	0	0	0	0.25	0.75	0.13	0	0
$scenario_2$	0	0	0	0	0.06	0.19	0	0.38	0.38	0.23	0	0.25
$scenario_3$	0.02	0.02	0.09	0.12	0.06	0.06	0.06	0.38	0.22	0.38	0.25	0.13

Results

To test the performance of ngsRelateV2, we use three simulated scenarios (see Simulations section) and compare it to ngsRelateV1 [11]. For every scenario, we generate 100 independent simulations for every combination of sequencing effort and number of segregating sites. In the first scenario, we compare two outbred cousins (Figure 1). As expected, both versions of ngsRelate find not only the correct level of relatedness, but also the correct estimates of the three relevant Jacquard coefficients (J_7, J_8, J_9). The second scenario also includes two cousins, but this time we have introduced inbreeding in one of the individuals. The parents of the inbred individual are related corresponding to a parent-child relation. In this scenario, even at low sequencing effort and only 10k sites, ngsRelateV2 correctly estimates the coefficients of relatedness and inbreeding; however, the estimates of the nine Jacquard coefficients are somewhat noisy, and at least 50k segregating sites are needed to increase the accuracy (Figure 2). The final scenario, being the most complex, includes the inbred individual from scenario two and another inbred cousin with its parents being related corresponding to a grandparent-grandchild relation. Interestingly, with such a complex pedigree, ngsRelateV2 still manages to recover the exact estimates for relatedness and individual inbreeding coefficients, even with only 10k segregating sites and a low sequencing depth (Figure 3). Similarly to the results from scenario two, accurate estimates of the nine Jacquard coefficients required increasing the number of informative sites and/or the sequencing effort. We also applied ngsRelateV2 to these three scenarios using a MAF cutoff on 0.05 (Supplementary Figure 1-3). We find that ngsRelateV2 recovers comparable accuracy with a MAF filter on 0.05.

We also applied ngsRelateV2 to real HTS data and compared the estimates to those obtained with ngsRelateV1. We used six pairwise related genomes, sequenced to low coverage (approximately 4X), from the LWK population generated as part of the 1000 Genomes Project [21]. We calculated genotype likelihoods of the related individuals, using ANGSD [18], at genomic sites with MAF in the LWK population on 0.05, summing up to 4.6m segregating sites. We not only show that ngsRelateV2 obtains comparable relatedness estimates to those obtained by ngsRelateV1, with this novel software, we also show that all the tested individuals show an inbreeding coefficient below 1% (Figure 4).

In extremely complicated pedigrees with symmetric inbreeding, such as multiple generations of full sibling mating, we find multiple global maxima where several combinations of the nine Jacquard coefficients, including the expected coefficients, are equally likely. Albeit observing such identifiability challenges, we, importantly, still find accurate relatedness estimates and individual inbreeding coefficients by summing the relevant Jacquard coefficients.

For every pair of individuals, ngsRelateV2 generates and outputs estimates of the nine Jacquard coefficients, the relatedness, the individual inbreeding coefficients as described above but also other combinations of the nine Jacquard coefficient: the kinship coefficient, fraternity, and the three summary statistics inbred relatedness, identity, and zygosity, suggested by Ackerman and colleagues [20]. It also produces the KING statistic [22] based on the two dimensional site frequency spectrum of pairs of individuals following the methodology in [17]. The latter statistics do not require population allele frequencies.

Table 4. Run statistics for 34 GB BCF file, as a function of number of cores. Shown is the memory requirement, wall-clock (actual runtime) and CPU time (both in hours), see also Figure 5.

Cores	Memory Usage	Wall-clock	CPU-time
1	45GB	85.59	87.14
2	46.9GB	41.38	81.39
4	49.3GB	23.36	89.03
8	54.2GB	11.88	88.69
16	63.5GB	6.30	89.73
32	83.2GB	3.39	87.81

Computational speed and memory requirements

To take advantage of the increasing number of cores of available on modern computers we employ a multilevel threading approach by both parallelizing both the file reading and the actual analysis. In Figure 5 we analyzed a semi-random dataset consisting of 135 samples mainly from the [23] publication. The input for the program was a 34 GB BCF file generated with standard bcftools with a liberal 164 mio number of SNP sites. We timed the actual runtime (wall-clock) and the CPU-time for varying number of cores (1,2,4,8,16,32) and noted the memory usage for each run, since allocating more cores for the process requires additional internal datastructures and does therefore also increases the memory requirements as seen by Table 4. From both the table and figure we observe a near linear correlation between the number of cores and the runtime with the CPU time remaining almost constant.

Conclusion

The tool presented in this technical note allows researchers to perform relatedness analysis for inbred individuals in a statistical framework that is especially suited for low coverage sequence data. The results show that the method performs well for estimating all nine coefficients, at least when the underlying pedigrees are not extremely complex. And even when the underlying pedigree is very complex, compound summaries of the output, like relatedness and inbreeding coefficients, will still be correct. The implementation is a fast multi threaded C++ program that can be directly applied to the most commonly used data files used for high throughput sequencing data.

Implementation Details

The program is implemented in a fast multithreaded c++ program and takes as input either genotype likelihood files and frequencies, bcf/vcf files as produced from standard tools such as GATK[24] or SAMtools [25], and binary-format plink files [3]. We also include an R implementation that we used for simulating data. Of note, the simulations generated in this study do not accounted for LD. In case of LD between genetic variants, the likelihood function becomes a composite likelihood function. The maximum likelihood estimate of such a function is consistent to that found with a likelihood function of independent sites [26].

The optimization follows the approach described in [11]. The optimization is an accelerated expectation maximization (EM) following the squared iterative approach in **S3** in [27] and is initialized with a random start point within the parameter space. The borders of the parameter space are manually examined after convergence. Since the EM algorithm is only guaranteed to find a local optimum, it is recommended to rerun with multiple different seeds though we note that we did not find an issue with multiple local optima in our examples.

Availability of source code and requirements

- Project name: ngsRelateV2
- Project home page: http://github.com/ANGSD/ngsRelate
- Operating system(s): Platform independent
- Programming language: C++
- Other requirements: htslib (only for parsing VCF/BCF files)
- License: GNU GPL (version 3)
- RRID: SCR_016588
- GigaDB: Snapshots of the code and other supporting data are available in the GigaScience repository, [28]

Declarations

List of abbreviations

IBD: Identity-by-descent. HTS: High-throughput sequencing.

Consent for publication

Not applicable.

Competing Interests

None.

Funding

KH is funded by the Danish National Research Foundation (DNRF94) and the Initiative d'Excellence Chaires d'attractivité, Université de Toulouse (OURASI); TSK by a grant from the Carlsberg Foundation (CF16-0913), Danish National Research Foundation Centre for GeoGenetics Funding, (DNRF 0094), Lundbeck Foundation GeoGenetics Centre for Brain, Disease, & Evolution grant number is: R302-2018-2155; IM by Independent Research Fund Denmark (DFF – 4090-00244); AM by an ERC Consolidator Grant LocalAdaptation 647787.

Authors' Contributions

TSK devised the model. It was first prototyped by PAA as part of a master project under the supervision of TSK and IM. KH implemented and ran all analyses. IM and AM devised test scenarios and improved early versions of the method. All authors wrote the article.

Acknowledgements

We want to thank the reviewers for their helpful comments.

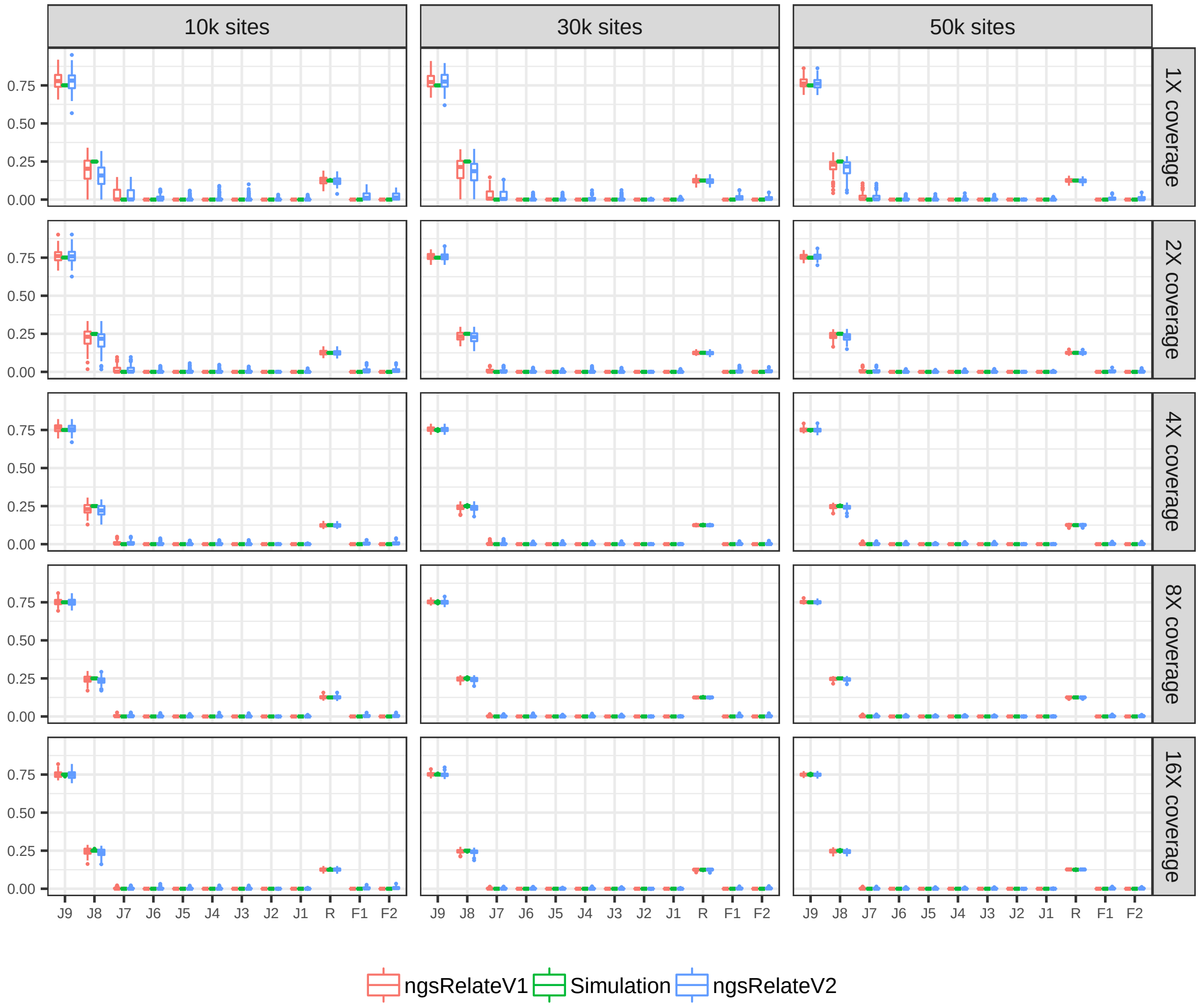

Figure 1. 100 independent simulations of two outbred cousins across variable sequencing depth and informative sites with a minor allele frequency cutoff on 10%. J_9 to J_1 refer to the nine Jacquard coefficients, R is the relatedness, finally, F_1 and F_2 refer to the individual inbreeding coefficients. Simulation (green) are the true values that we compare ngsRelateV1 (red) and the new program ngsRelateV2 (blue) against.

References

1. Weir BS, Anderson AD, Hepler AB. Genetic relatedness analysis: modern data and new challenges. Nat Rev Genet 2006 Oct;7(10):771–780.
2. Cotterman C. Relatives and human genetic analysis. The Scientific Monthly 1941;53(3):227–234.
3. Purcell S, Neale B, Todd-Brown K, Thomas L, Ferreira MA, Bender D, et al. PLINK: a tool set for whole-genome association and population-based linkage analyses. Am J Hum Genet 2007 Sep;81(3):559–575.
4. Thompson EA. The estimation of pairwise relationships. Ann Hum Genet 1975 Oct;39(2):173–188.
5. Manichaikul A, Mychaleckyj JC, Rich SS, Daly K, Sale M, Chen WM. Robust relationship inference in genome-wide association studies. Bioinformatics 2010 Nov;26(22):2867–2873.
6. Ritland K. Estimators for pairwise relatedness and individual inbreeding coefficients. Genetical Research 1996;67(2):175–185.
7. Milligan BG. Maximum-likelihood estimation of relatedness. Genetics 2003 Mar;163(3):1153–1167.
8. Anderson AD, Weir BS. A maximum-likelihood method for the estimation of pairwise relatedness in structured populations. Genetics 2007 May;176(1):421–440.
9. Wang J. COANCESTRY: a program for simulating, estimating and analysing relatedness and inbreeding coefficients. Mol Ecol Resour 2011 Jan;11(1):141–145.
10. Kuhn JMM, Jakobsson M, Gunther T. Estimating genetic kin relationships in prehistoric populations. PLoS ONE 2018;13(4):e0195491.
11. Korneliussen TS, Moltke I. NgsRelate: a software tool for estimating pairwise relatedness from next-generation sequencing data. Bioinformatics 2015 Dec;31(24):4009–4011.
12. Hedrick PW, Lacy RC. Measuring Relatedness between Inbred Individuals. Journal of Heredity 2015;106(1):20–25.
13. Vieira FG, Fumagalli M, Albrechtsen A, Nielsen R. Estimating inbreeding coefficients from NGS data: Impact on genotype calling and allele frequency estimation. Genome Res 2013 Nov;23(11):1852–1861.
14. Ko A, Nielsen R. Composite likelihood method for inferring local pedigrees. PLoS Genet 2017 Aug;13(8):e1006963.
15. Sun M, Jobling MA, Taliun D, Pramstaller PP, Egeland T, Sheehan NA. On the use of dense SNP marker data for the identification of distant relative pairs. Theor Popul Biol 2016 Feb;107:14–25.

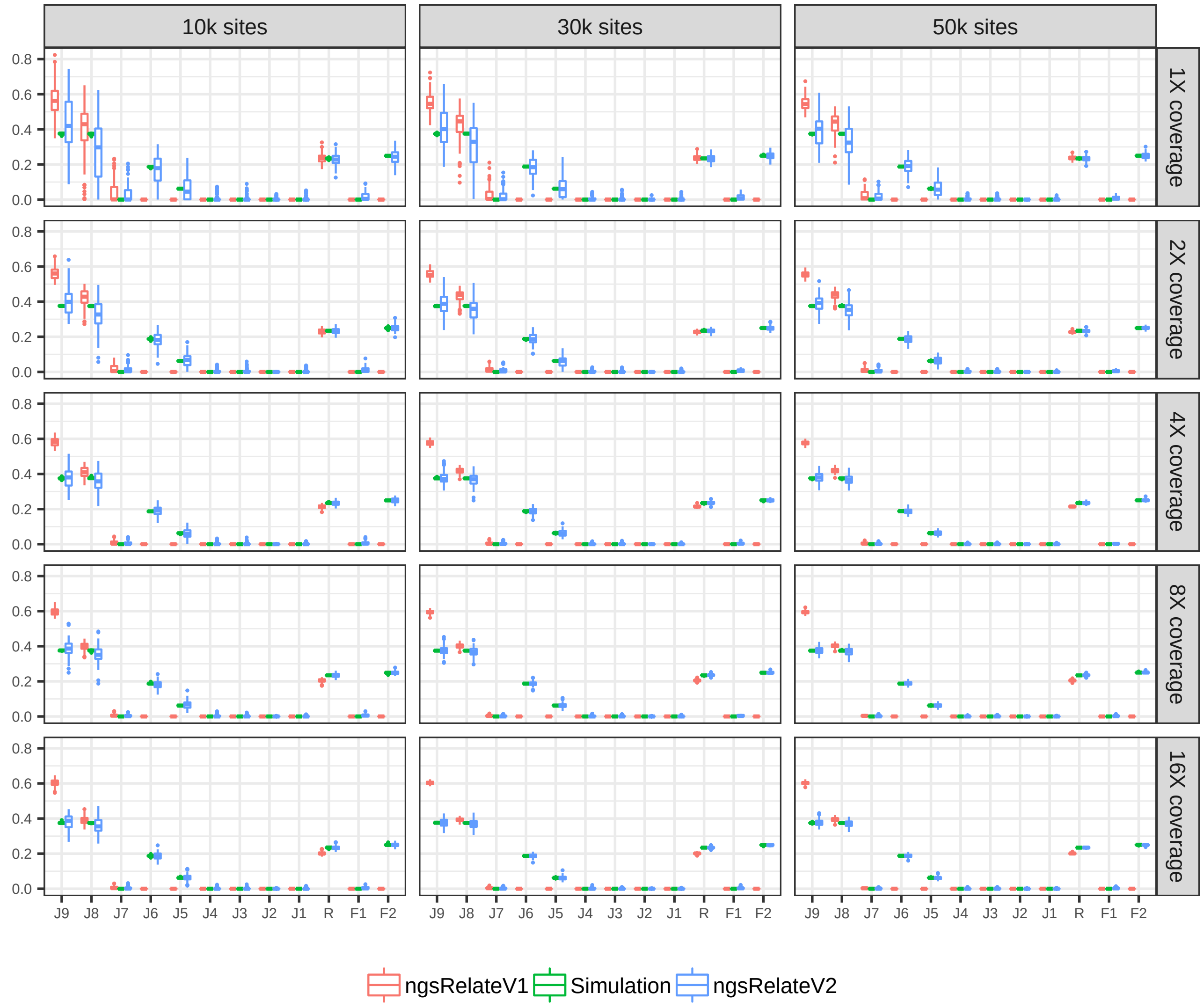

Figure 2. 100 independent simulations of two cousins, with one individual being inbred, across variable sequencing depth and segregating sites with a minor allele frequency cutoff on 10%. J_9 to J_1 refer to the nine Jacquard coefficients, R is the relatedness, finally, F_1 and F_2 refer to the individual inbreeding coefficients. Simulation (green) are the true values that we compare ngsRelateV1 (red) and the new program ngsRelateV2 (blue) against.

16. Csűrös M. Non-identifiability of identity coefficients at biallelic loci. Theoretical population biology 2014;92:22–29.
17. Waples RK, Albrechtsen A, Moltke I. Allele frequency-free inference of close familial relationships from genotypes or low depth sequencing data. Molecular ecology 2018;.
18. Korneliussen TS, Albrechtsen A, Nielsen R. ANGSD: Analysis of Next Generation Sequencing Data. BMC Bioinformatics 2014 Nov;15(1):356.
19. Jacquard A. The genetic structure of populations, vol. 5. Springer; 1974.
20. Ackerman MS, Johri P, Spitze K, Xu S, Doak TG, Young K, et al. Estimating Seven Coefficients of Pairwise Relatedness Using Population-Genomic Data. Genetics 2017 05;206(1):105–118.
21. The 1000 Genomes Project Consortium, et al. An integrated map of genetic variation from 1,092 human genomes. Nature 2012;491(7422):56.
22. Manichaikul A, Mychaleckyj JC, Rich SS, Daly K, Sale M, Chen WM. Robust relationship inference in genome-wide association studies. Bioinformatics 2010 Nov;26(22):2867–2873.
23. de Barros Damgaard P, Martiniano R, Kamm J, Moreno-Mayar JV, Kroonen G, Peyrot M, et al. The first horse herders and the impact of early Bronze Age steppe expansions into Asia. Science 2018 06;360(6396).
24. McKenna A, Hanna M, Banks E, Sivachenko A, Cibulskis K, Kernytsky A, et al. The Genome Analysis Toolkit: a MapReduce framework for analyzing next-generation DNA sequencing data. Genome Res 2010 Sep;20(9):1297–1303.
25. Li H, Handsaker B, Wysoker A, Fennell T, Ruan J, Homer N, et al. The Sequence Alignment/Map format and SAMtools. Bioinformatics 2009 Aug;25(16):2078–2079.
26. Lindsay BG. Composite likelihood methods. Contemporary mathematics 1988;80(1):221–239.
27. Varadhan R, Roland C. Simple and globally convergent methods for accelerating the convergence of any EM algorithm. Scandinavian Journal of Statistics 2008;35(2):335–353.
28. Hanghøj K, Moltke I, Andersen PA, Manica A, Korneliussen TS. Supporting data for "Fast and accurate relatedness estimation from high throughput sequencing data in the presence of inbreeding". GigaDB 2019;http://dx.doi.org/10.5524/100562.

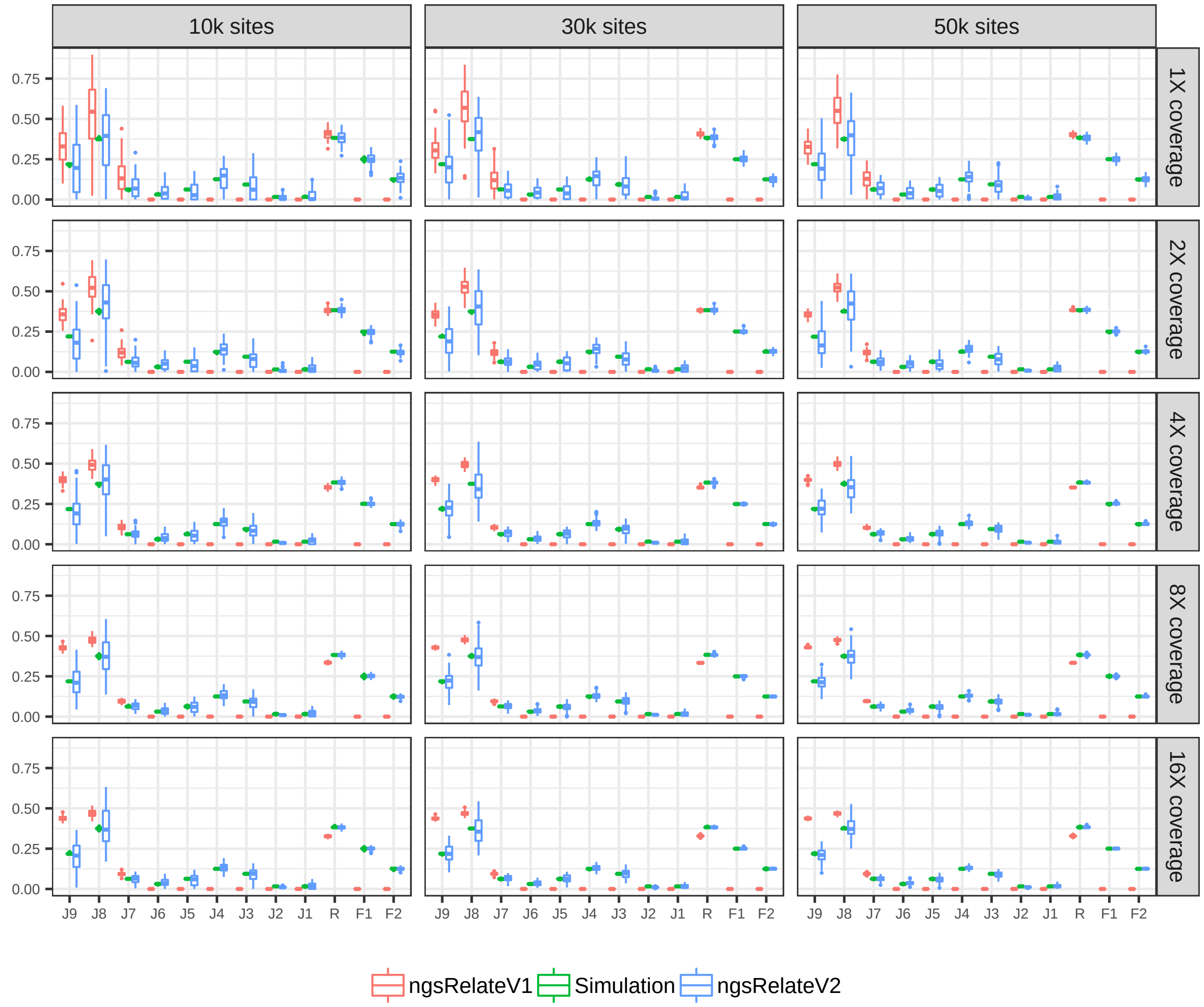

Figure 3. 100 independent simulations of two cousins, both being inbred, across variable sequencing depth and segregating sites with a minor allele frequency cutoff on 10%. J_9 to J_1 refer to the nine Jacquard coefficients, R is the relatedness, finally, F_1 and F_2 refer to the individual inbreeding coefficients. Simulation (green) are the true values that we compare ngsRelateV1 (red) and the new program ngsRelateV2 (blue) against.

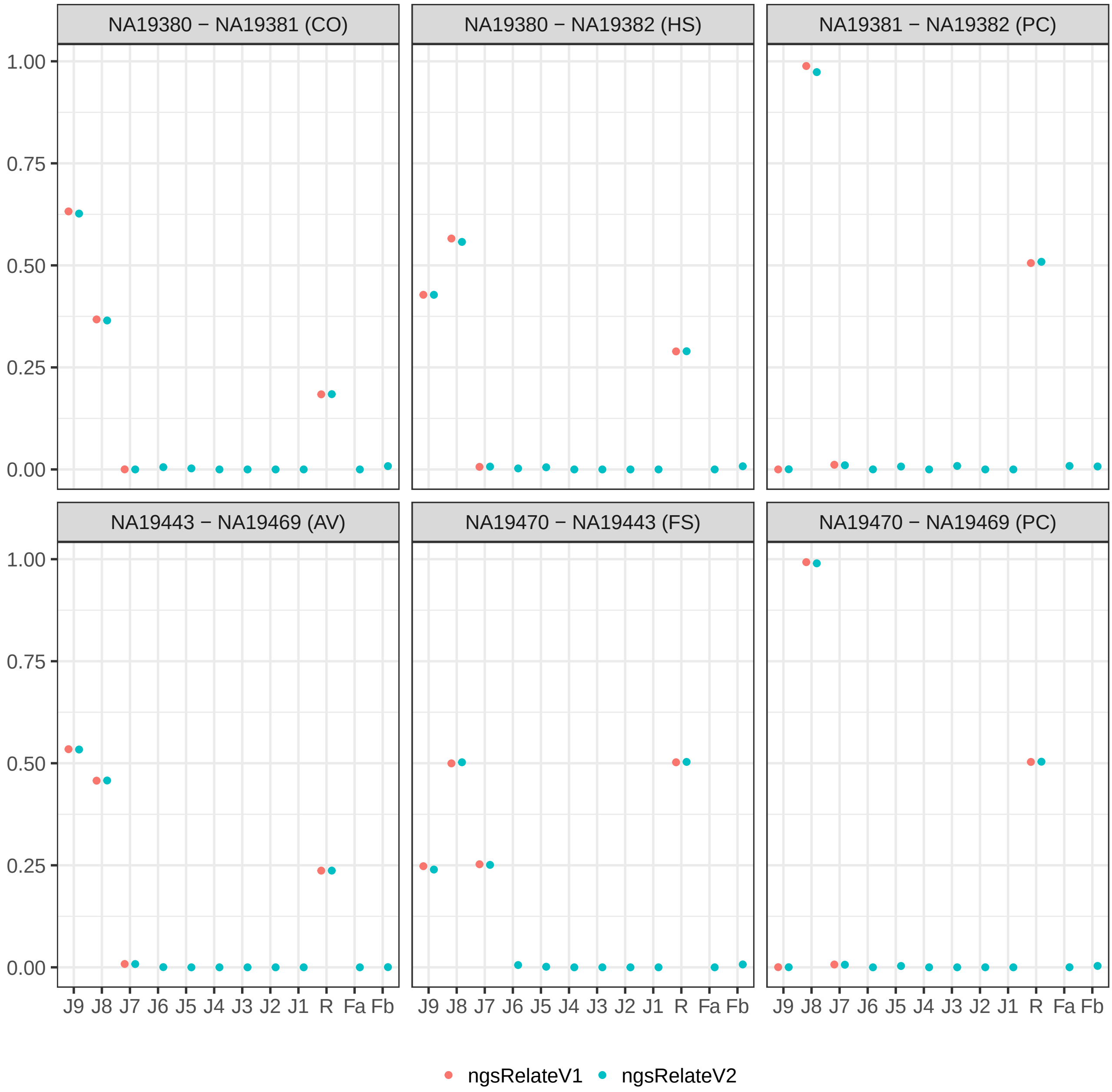

Figure 4. Estimated Jacquard coefficients from six pairs of related individuals. The estimates are based on low-depth NGS data from the 1000 Genomes Project using ngsRelateV1 and ngsRelateV2. J_9 to J_1 refer to the nine Jacquard coefficients, R is the relatedness, finally, F_1 and F_2 refer to the individual inbreeding coefficients. CO: Cousins, HS: Half Siblings, PC: Parent-Child, AV: Avuncular, FS: Full Siblings.

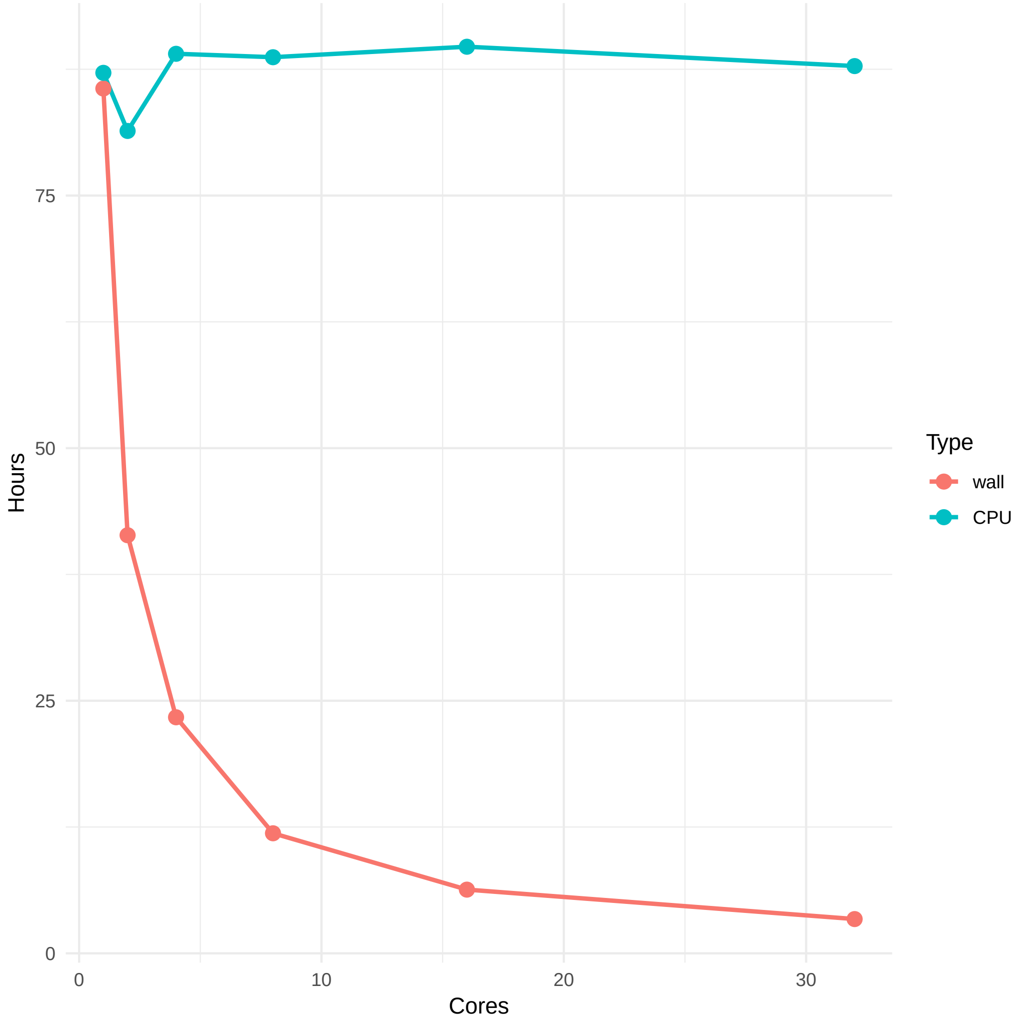

Figure 5. Running times for ngsRelateV2 on a dataset with 135 individuals 164mio possible SNP sites. Blue line is the overall CPU usage across all threads allocated to the main process. Redline being the runtime for the process to finish. The actual values along with memory usage can be found in Table 4
.

Supplementary Material

Click here to access/download
Supplementary Material
IBD_GIGASCIENCE-4.pdf
