# Supplementary material for: Fast and accurate relatedness estimation from high-throughput sequencing data in the presence of inbreeding
Source: Gigascience. 2019 Apr 30;8(5):giz034. doi: 10.1093/gigascience/giz034 (PMC6488770; doi:10.1093/gigascience/giz034)
Supplement: Supplement_Figures.pdf [file giz034_supplement_figures.pdf]

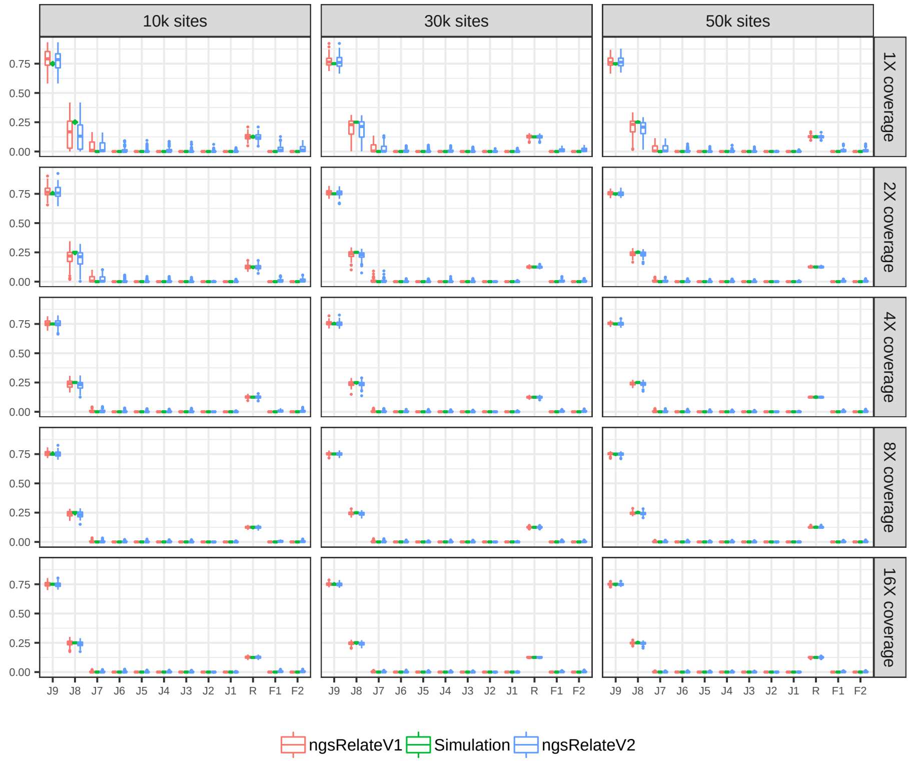

Supplementary Figure 1: 100 independent simulations of two out-bred cousins across variable sequencing depth and informative sites with a minor allele frequence cutoff on 5%. J_9 to J_1 refer to the nine Jacquard coefficients, R is the relatedness, finally, F_1 and F_2 refer to the individual inbreeding coefficients. Simulation (green) are the true values that we compare ngsRelateV1 (red) and the new program ngsRelateV2 (blue) against.

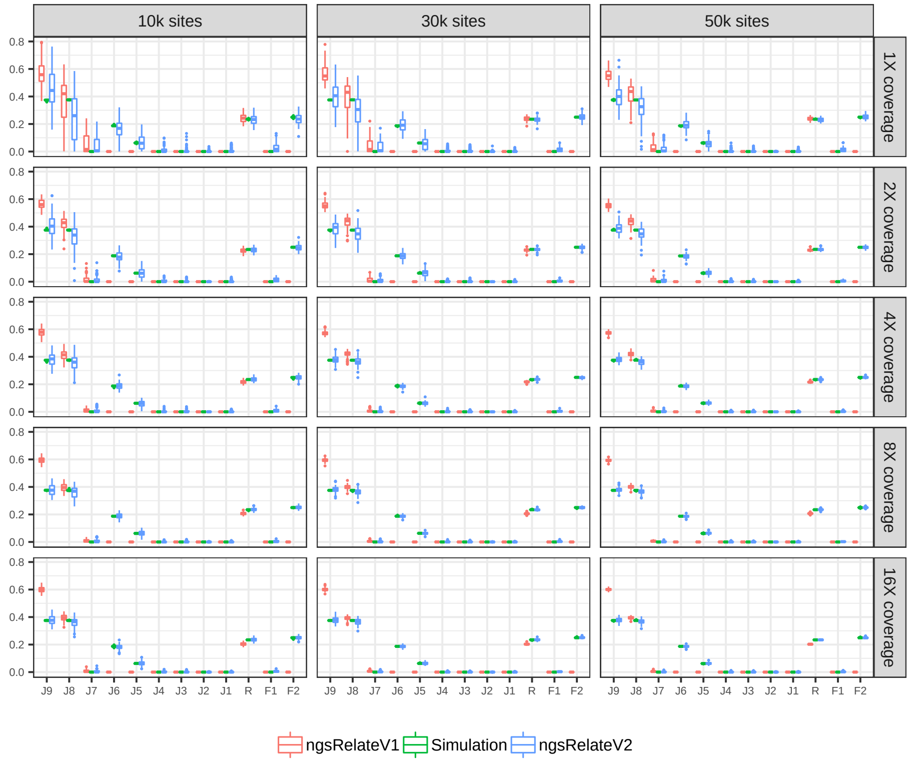

Supplementary Figure 2: 100 independent simulations of two cousins, with one individual being inbred, across variable sequencing depth and segregating sites with a minor allele frequence cutoff on 5%. J_9 to J_1 refer to the nine Jacquard coefficients, R is the relatedness, finally, F_1 and F_2 refer to the individual inbreeding coefficients. Simulation (green) are the true values that we compare ngsRelateV1 (red) and the new program ngsRelateV2 (blue) against.

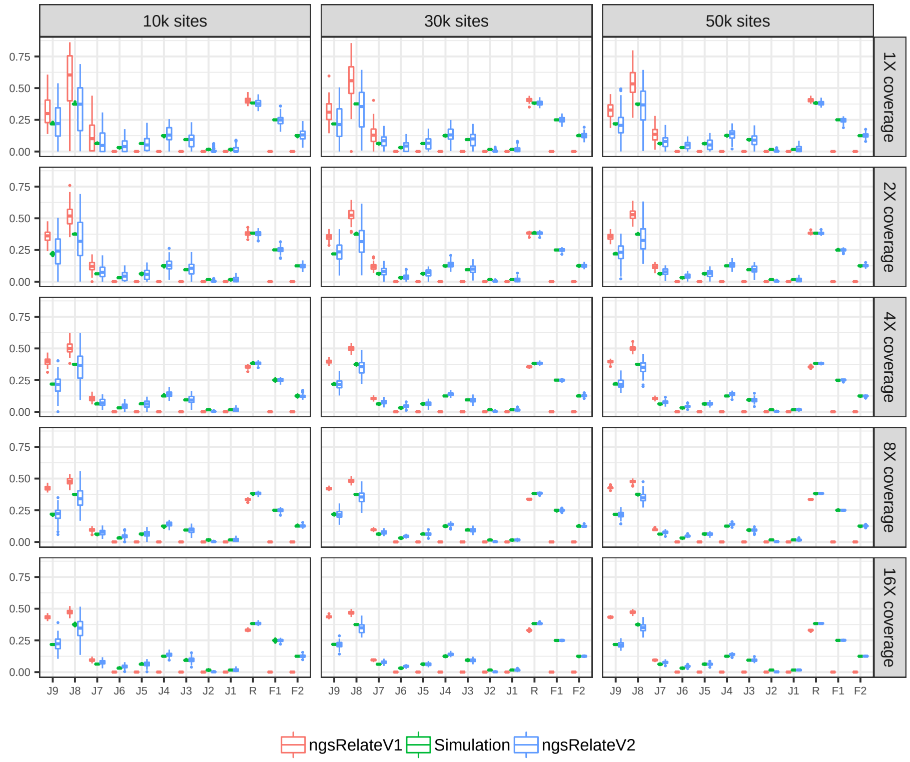

Supplementary Figure 3: 100 independent simulations of two cousins, both being inbred, across variable sequencing depth and segregating sites with a minor allele frequence cutoff on 5%. J_9 to J_1 refer to the nine Jacquard coefficients, R is the relatedness, finally, F_1 and F_2 refer to the individual inbreeding coefficients. Simulation (green) are the true values that we compare ngsRelateV1 (red) and the new program ngsRelateV2 (blue) against.
