# Supplementary material for: Fast and accurate relatedness estimation from high-throughput sequencing data in the presence of inbreeding
Source: Gigascience. 2019 Apr 30;8(5):giz034. doi: 10.1093/gigascience/giz034 (PMC6488770; doi:10.1093/gigascience/giz034)
Supplement: Response-to-Reviewer-Comments-Revision-1.pdf [file giz034_response-to-reviewer-comments-revision-1.pdf]

Author's Response To Reviewer Comments

Close

We have fixed all comments from reviewer, which we found very useful and helpful.

Additionally:
We have further extended ngsRelateV2 to take plink files as input.
We have also added a figure to show that the threading works as expected along with runtime estimates. (Figure 5) and a table (Table 4) that also shows memory usage for different number of threads allocated to the program
Implementation wise we have added the possibility of doing bootstrap replicates along with performing multiple optimizations on the fly to circumvent the issue of local optima
Added missing funding bodies.
Added the reviewers comments in the acknowledgement
Added an additional coauthor. The coauthor that has been added was working on this as part of his MSc project.
We have added a citation to the associated gigadb entry

Reviewer reports:
Reviewer #2: The authors have updated their manuscript, taking most of the suggestions of the reviewers satisfactorily into account. I have some additional comments, mostly minor, that may be of help for the authors to further improve their manuscript.

Abstract: "In the case low coverage" --> "in the case of low coverage"
DONE
Pg. 2, l. 4. The authors may wish to add "respectively, and known as Cotterman's coefficients (Cotterman, 1941)."
DONE
Pg. 2, l. 28. "as well as inbreeding coefficient" --> "as well as inference of the inbreeding coefficients"
DONE
Pg. 2, l. 6, 2nd col. "population frequencies" --> "population allele frequencies"
DONE
Pg. 2, l. 30, 2nd col. "Bernoulli trials" --> "independent Bernoulli trails"
DONE
Pg. 2, l. 30, 2nd col. "given site." The authors may wish to add: "given site, implying the data is generated under the assumption of Hardy-Weinberg equilibrium."
DONE
Pg. 3, l. 8: "estimated from" --> "estimated by"
DONE
Pg. 3, l. 18: J7; the "7" should be a subscript.
DONE
Pg. 3, l. 33: It is not clear what is meant with "confident estimates". Improved estimates?
We have changed it to “accurate estimates”

Pg. 3, l. 60: Supplementary figure 4. This is an important application showing that the proposed estimation procedure seems to work well in practice. I suggest the figure not to be supplementary, but part of the main manuscript. The documented relationships of the six panels should be clearly indicated, for instance by putting (PO), (FS) or whatever corresponds in the banner of each panel behind the identifiers of each pair. Documented relationships could be mentioned in the text or the caption of the figure as well.

We have moved supplementary figure 4 to the main manuscript and added the pairwise relationships to the figure.

The authors have apparently been a bit careless in their update of the bibliography. Please address the

following issues:

16: the author name is misspelled.
DONE
17. This book is not from 2015, but from 1974. Please check out the correct reference.
DONE
19. The author, the 1000G Consortium, is not correctly spelled out.
DONE
25. Surnames not fully in uppercase.
DONE

Close
