# Supplementary material for: Fast and accurate relatedness estimation from high-throughput sequencing data in the presence of inbreeding
Source: Gigascience. 2019 Apr 30;8(5):giz034. doi: 10.1093/gigascience/giz034 (PMC6488770; doi:10.1093/gigascience/giz034)
Supplement: Response-to-Reviewer-Comments_Original-Submission.pdf [file giz034_response-to-reviewer-comments_original-submission.pdf]

Author's Response To Reviewer Comments

Close

GIGA-D-18-00338
Fast and accurate relatedness estimation from high throughput sequencing data in the presence of inbreeding
Kristian Hanghoej, msc; Ida Moltke, PhD; Andrea Manica, PhD; Thorfinn Sand Korneliussen, Ph.D
GigaScience

Dear Dr Korneliussen,

Your manuscript "Fast and accurate relatedness estimation from high throughput sequencing data in the presence of inbreeding" (GIGA-D-18-00338) has been assessed by our reviewers. Although it is of interest, we are unable to consider it for publication in its current form. The reviewers have raised a number of points which we believe would improve the manuscript and may allow a revised version to be published in GigaScience.

Their reports are below.

Please address all of the reviewers' points, but I'd like to highlight two concerns that are really crucial for the potential acceptance of a revised version:

1. Both reviewers mention that real data should be used to demonstrate the tool's capabilities in addition to your simulations. This recommendation is in line with the expected standard for our "Technical Notes", and I urge you to present a use case with real data.

We agree with the reviewers and added six pairwise comparisons of related individuals. ngsRelateV2 obtains comparable relatedness estimates to those obtained with ngsRelateV1. We also show that ngsRelateV2 in a single run obtains inbreeding coefficient for all the analyzed individuals. See also comment 7 from reviewer 2. We have screened the literature for populations with a pair of related individuals that also show a nonzero level of inbreeding. This includes a rice data set (doi:10.1101/gr.157388.113) and a population of mountain gorillas (DOI:10.1126/science.aaa3952). However, we have not been able to identify a data set, that full fill both of the above-mentioned criteria. If you are aware of such dataset, we will be pleased to apply ngsRelateV2 to it.

2. Related to the first point, reviewer 2 wonders how the tool performs with fewer restrictions, and in a more realistic setting. Please carefully explore and discuss this aspect in the revised manuscript, following the guidance of our reviewers.

As described in our reply to the first reviewers concern (comment 5), we have now performed additional simulations with fewer restrictions. Specifically, as requested by the reviewer, we have performed all simulations based on a minor allele frequency filter on 0.05 as well. These additional simulations demonstrate that ngsRelateV2 also manages to obtain the correct related and inbreeding estimates with a less stringent filtering.

In addition, please register the new software application in the SciCrunch.org database to receive a RRID (Research Resource Identification Initiative ID) number, and include this in your manuscript. This will facilitate tracking, reproducibility and re-use of your tool.
We have now registered the software on scicrunch and have obtained the RRID SCR_016588 which we have included in the manuscript section 'Availability'.

Please also clarify in the manuscript which open licence is applied for your code (the statement seems ambiguous at the moment).

We have set the licence to GPL v3.

If you are able to fully address these points, we would encourage you to submit a revised manuscript to GigaScience. Once you have made the necessary corrections, please submit online at:

https://giga.editorialmanager.com/

If you have forgotten your username or password please use the "Send Login Details" link to get your login information. For security reasons, your password will be reset.

Please include a point-by-point within the 'Response to Reviewers' box in the submission system. Please ensure you describe additional experiments that were carried out and include a detailed rebuttal of any criticisms or requested revisions that you disagreed with. Please also ensure that your revised manuscript conforms to the journal style, which can be found in the Instructions for Authors on the journal homepage.

The due date for submitting the revised version of your article is 06 Jan 2019.

I look forward to receiving your revised manuscript soon.

Best wishes,

Hans Zauner
GigaScience
www.gigasciencejournal.com

Reviewer reports:

Reviewer #1: In this MS, the authors introduce a new method to estimate the 9 condensed IBD coefficients from high throughput sequencing data (with genotyping errors). It is implemented in the software package ngsRelateV2, which is a major revision of ngsRelateV1. The new software can estimate inbreeding, and estimate relatedness correctly in the presence of inbreeding. The authors demonstrated the accuracy of the method by analyzing some simulated data. It is a valuable paper worth publishing.

I have the following comments for the authors to consider in revision.

1. It is claimed that the 9 IBD configurations cannot be identified and the 9 corresponding IBD coefficients cannot be inferred from biallelic data, such as SNPs. The reference is
Csűrös, M., 2014. Non-identifiability of identity coefficients at biallelic loci. Theoretical population biology, 92, pp.22-29.
What do you think?

Thank you for pointing out this paper. We observed this problem when working on simulations of complex pedigress with high levels of inbreeding. Csűrös (2014) suggests additional quantities (such as two-out-of-three IBD) which are unambiguous, and we have now included them in out output.(see the wiki documentation for a complete list of summary statistics implemented in ngsRelateV2, https://github.com/angsd/ngsrelate). In addition to these we have added an additional approach for estimating relatedness based on the 2 dimensional site frequency spectrum which is therefore free of assumption of population frequencies. We therefore envision that that software and tool encapsulates a wide variety of existing methods and approaches for estimating relatedness.

2. You missed a relevant paper,

Wang, J., 2007. Triadic IBD coefficients and applications to estimating pairwise relatedness. Genetics Research, 89(3), pp.135-153.

In this paper, a likelihood method was proposed to estimate the 9 condensed IBD coefficients from marker (microsatellites or SNPs) data with possible genotyping errors. The method is implemented in the software Coancestry (the inbreeding model).
Yes we agree completely this paper is very relevant to cite, and we have added the proper citation in the introduction, as reference [8].

3. There are now many published NGS data. Why not analysing a real dataset to demonstrate the new method? Real data are much more complicated than simulated data. But your method and software is for applications to real data, not simulated data.

We agree with the reviewer (See answer 7).

4. It is unclear to me which algorithm is used to maximize the likelihood for the estimation of the IBD coefficients. How reliable and how fast is the algorithm? Do you need some initial IBD coefficient values to start searching the ML estimates? How do you choose the initial values? Are the results reliant on initial points? These need to be clarified (though not detailed to save space).

We have now clarified the algorithmic details regarding the optimization in a new subsection under "implementation details".

Reviewer #2: This paper presents a computer program, ngsRelateV2, for the estimation of relatedness parameters from high-throughput sequencing data. The authors use a maximum likelihood based approach to estimate 9 Jacquard coefficients, which is more refined than most empirical studies that use 3 coefficients only. They show the new version of their method can account for inbreeding, and provides better estimates of the Jacquard coefficients in comparison with an earlier version of their method that did not account for inbreeding. This is an interesting and well written paper. I have some major and minor concerns given below.

Major issues

5. The simulations of the authors use several restrictions, which maybe could be relaxed, or should at least be dealt with in more detail. A MAF filter of 0.10 is applied to the founder generation, which is not very realistic, as low MAF variants are in practice more common. What do the authors do when they estimate the Jacquard coefficients by ML? Do they also apply the 0.10 filter to the data prior to estimation? If so, a filter at data generation stage may not be needed. I suggest the authors to repeat the simulations with no and lower (0.05) MAF filter and report the corresponding results, as this will give more information on the performance of their method.

We agree that a MAF filter of 0.05 is probably more commonly used and thus more realistic. We have therefore now performed additional simulations and performed analyses with a 5% allele frequency cutoff (Supplementary figure 1-3). We find that this has no effect on the accuracy of ngsRelate. We have also committed the R script to generate the simulations analyzed in this study to the github (https://github.com/ANGSD/NgsRelate/blob/master/make_simulations.R). The model is based on the existence of population frequencies for diallelic sites, effectively invariant sites will not bring information to the likelihood function.

6. The simulations are carried out in a site-wise manner, by sampling sites independently. Real inheritance is chromosome-wise, with the corresponding LD structure and recombination process. All of this is ignored in the simulations. This implies the authors assume there are as many chromosomes as simulated sites, which is unrealistic. Accounting for all this in the simulations is complicated, but it could be done. The authors should at least address the limitations of their simulations in the discussion section.

We agree that we do not model LD and have now clarified this further in the text (simulation section and implementation details).

7. The authors only work with simulated data, using only 3 very specific scenarios for an FC relationship. They do not give a single empirical data example, which is a serious omission. The analysis of some empirical data could greatly strengthen the paper. There is plenty of empirical data available (e.g. from the 1000 genomes project), and I suggest the authors to analyse all pairs of one population of the 1000G project, and to compare their results with those documented by other researchers (Pemberton et al, 2010).

As suggested by the reviewer, we have analyzed low-coverage NGS data from the 1000 genome project where the relatedness is known. Specifically, we have tested six pairs known to be related in the Luhya in Webuye, Kenya (LWK) population. ngsRelateV2 find estimates overlapping those found with ngsRelateV1. We also show that these individuals display an inbreeding level less than 1%.

8. The authors refer to their results as "confidence intervals" (p.2 l.61), but these are boxplots of the values of 100 simulations, which is something very different. Please correct the terminology.

This is an error that has been fixed.

Minor issues

9. Legend figure 2. Acquard coefficients -- Jacquard coefficients.
Yes, this has been edited. Thank you for catching it.

References:

Pemberton T, Wang C, Li J, Rosenberg N. (2010) Inference of unexpected genetic relatedness among individuals in HapMap Phase III. American Journal of Human Genetics. 87:457-464.

--
Please also take a moment to check our website at https://giga.editorialmanager.com/l.asp?i=45544&l=S42OI455 for any additional comments that were saved as attachments. Please note that as GigaScience has a policy of open peer review, you will be able to see the names of the reviewers.

In compliance with data protection regulations, please contact the publication office if you would like to have your personal information removed from the database.

Close
