# Supplementary material for: Fast and accurate relatedness estimation from high-throughput sequencing data in the presence of inbreeding
Source: Gigascience. 2019 Apr 30;8(5):giz034. doi: 10.1093/gigascience/giz034 (PMC6488770; doi:10.1093/gigascience/giz034)
Supplement: Reviewer-1-Report-Original-Submission -- Jinliang Wang — 9/23/2018 Reviewed [file giz034_reviewer-1-report-original-submission.pdf]

Reviewer Report

Title: Fast and accurate relatedness estimation from high throughput sequencing data in the presence of inbreeding

Version: Original Submission **Date:** 9/23/2018

Reviewer name: Jinliang Wang

Reviewer Comments to Author:

In this MS, the authors introduce a new method to estimate the 9 condensed IBD coefficients from high throughput sequencing data (with genotyping errors). It is implemented in the software package ngsRelateV2, which is a major revision of ngsRelateV1. The new software can estimate inbreeding, and estimate relatedness correctly in the presence of inbreeding. The authors demonstrated the accuracy of the method by analyzing some simulated data. It is a valuable paper worth publishing.
I have the following comments for the authors to consider in revision.
1. It is claimed that the 9 IBD configurations cannot be identified and the 9 corresponding IBD coefficients cannot be inferred from biallelic data, such as SNPs. The reference is
CsÅ±rÃ¶s, M., 2014. Non-identifiability of identity coefficients at biallelic loci. Theoretical population biology, 92, pp.22-29.
What do you think?
2. You missed a relevant paper,
Wang, J., 2007. Triadic IBD coefficients and applications to estimating pairwise relatedness. Genetics Research, 89(3), pp.135-153.
In this paper, a likelihood method was proposed to estimate the 9 condensed IBD coefficients from marker (microsatellites or SNPs) data with possible genotyping errors. The method is implemented in the software Coancestry (the inbreeding model).
3. There are now many published NGS data. Why not analysing a real dataset to demonstrate the new method? Real data are much more complicated than simulated data. But your method and software is for applications to real data, not simulated data.
4. It is unclear to me which algorithm is used to maximize the likelihood for the estimation of the IBD coefficients. How reliable and how fast is the algorithm? Do you need some initial IBD coefficient values to start searching the ML estimates? How do you choose the initial values? Are the results reliant on initial points? These need to be clarified (though not detailed to save space).

Level of Interest

Please indicate how interesting you found the manuscript: Choose an item.

Quality of Written English

Please indicate the quality of language in the manuscript: Choose an item.

Declaration of Competing Interests

Please complete a declaration of competing interests, considering the following questions:

- Have you in the past five years received reimbursements, fees, funding, or salary from an organisation that may in any way gain or lose financially from the publication of this manuscript, either now or in the future?
- Do you hold any stocks or shares in an organisation that may in any way gain or lose financially from the publication of this manuscript, either now or in the future?
- Do you hold or are you currently applying for any patents relating to the content of the manuscript?
- Have you received reimbursements, fees, funding, or salary from an organization that holds or has applied for patents relating to the content of the manuscript?
- Do you have any other financial competing interests?
- Do you have any non-financial competing interests in relation to this paper?

If you can answer no to all of the above, write 'I declare that I have no competing interests' below. If your reply is yes to any, please give details below.

I declare that I have no competing interests

I agree to the open peer review policy of the journal. I understand that my name will be included on my report to the authors and, if the manuscript is accepted for publication, my named report including any attachments I upload will be posted on the website along with the authors' responses. I agree for my report to be made available under an Open Access Creative Commons CC-BY license (http://creativecommons.org/licenses/by/4.0/). I understand that any comments which I do not wish to be included in my named report can be included as confidential comments to the editors, which will not be published.

I agree to the open peer review policy of the journal

To further support our reviewers, we have joined with Publons, where you can gain additional credit to further highlight your hard work (see: https://publons.com/journal/530/gigascience). On publication of this paper, your review will be automatically added to Publons, you can then choose whether or not to claim your Publons credit. I understand this statement.

Yes Choose an item.
