# Supplementary material for: Fast and accurate relatedness estimation from high-throughput sequencing data in the presence of inbreeding
Source: Gigascience. 2019 Apr 30;8(5):giz034. doi: 10.1093/gigascience/giz034 (PMC6488770; doi:10.1093/gigascience/giz034)
Supplement: Reviewer-2-Report-Original-Submission -- Jan Graffelman — 10/8/2018 Reviewed [file giz034_reviewer-2-report-original-submission.pdf]

Reviewer Report

Title: Fast and accurate relatedness estimation from high throughput sequencing data in the presence of inbreeding

Version: Original Submission **Date:** 10/8/2018

Reviewer name: Jan Graffelman

Reviewer Comments to Author:

This paper presents a computer program, ngsRelateV2, for the estimation of relatedness parameters from high-throughput sequencing data. The authors use a maximum likelihood based approach to estimate 9 Jacquard coefficients, which is more refined than most empirical studies that use 3 coefficients only. They show the new version of their method can account for inbreeding, and provides better estimates of the Jacquard coefficients in comparison with an earlier version of their method that did not account for inbreeding. This is an interesting and well written paper. I have some major and minor concerns given below.

Major issues

The simulations of the authors use several restrictions, which maybe could be relaxed, or should at least be dealt with in more detail. A MAF filter of 0.10 is applied to the founder generation, which is not very realistic, as low MAF variants are in practice more common. What do the authors do when they estimate the Jacquard coefficients by ML? Do they also apply the 0.10 filter to the data prior to estimation? If so, a filter at data generation stage may not be needed. I suggest the authors to repeat the simulations with no and lower (0.05) MAF filter and report the corresponding results, as this will give more information on the performance of their method.

The simulations are carried out in a site-wise manner, by sampling sites independently. Real inheritance is chromosome-wise, with the corresponding LD structure and recombination process. All of this is ignored in the simulations. This implies the authors assume there are as many chromosomes as simulated sites, which is unrealistic. Accounting for all this in the simulations is complicated, but it could be done. The authors should at least address the limitations of their simulations in the discussion section.

The authors only work with simulated data, using only 3 very specific scenarios for an FC relationship. They do not give a single empirical data example, which is a serious omission. The analysis of some empirical data could greatly strengthen the paper. There is plenty of empirical data available (e.g. from the 1000 genomes project), and I suggest the authors to analyse all pairs of one population of the 1000G project, and to compare their results with those documented by other researchers (Pemberton et al, 2010).

The authors refer to their results as "confidence intervals" (p.2 l.61), but these are boxplots of the values of 100 simulations, which is something very different. Please correct the terminology.

Minor issues

Legend figure 2. Acquard coefficients -- Jacquard coefficients.

References:

Pemberton T, Wang C, Li J, Rosenberg N. (2010) Inference of unexpected genetic relatedness among individuals in HapMap Phase III. American Journal of Human Genetics. 87:457-464.

Level of Interest

Please indicate how interesting you found the manuscript: Choose an item.

Quality of Written English

Please indicate the quality of language in the manuscript: Choose an item.

Declaration of Competing Interests

Please complete a declaration of competing interests, considering the following questions:

- Have you in the past five years received reimbursements, fees, funding, or salary from an organisation that may in any way gain or lose financially from the publication of this manuscript, either now or in the future?
- Do you hold any stocks or shares in an organisation that may in any way gain or lose financially from the publication of this manuscript, either now or in the future?
- Do you hold or are you currently applying for any patents relating to the content of the manuscript?
- Have you received reimbursements, fees, funding, or salary from an organization that holds or has applied for patents relating to the content of the manuscript?
- Do you have any other financial competing interests?
- Do you have any non-financial competing interests in relation to this paper?

If you can answer no to all of the above, write 'I declare that I have no competing interests' below. If your reply is yes to any, please give details below.

I declare that I have no competing interests

I agree to the open peer review policy of the journal. I understand that my name will be included on my report to the authors and, if the manuscript is accepted for publication, my named report including any attachments I upload will be posted on the website along with the authors' responses. I agree for my report to be made available under an Open Access Creative Commons CC-BY license (http://creativecommons.org/licenses/by/4.0/). I understand that any comments which I do not wish to be included in my named report can be included as confidential comments to the editors, which will not be published.

I agree to the open peer review policy of the journal

To further support our reviewers, we have joined with Publons, where you can gain additional credit to further highlight your hard work (see: https://publons.com/journal/530/gigascience). On publication of this paper, your review will be automatically added to Publons, you can then choose whether or not to claim your Publons credit. I understand this statement.

Yes Choose an item.
