# Supplementary material for: Fast and accurate relatedness estimation from high-throughput sequencing data in the presence of inbreeding
Source: Gigascience. 2019 Apr 30;8(5):giz034. doi: 10.1093/gigascience/giz034 (PMC6488770; doi:10.1093/gigascience/giz034)
Supplement: Reviewer-2-Report-Revision-1 -- Jan Graffelman — 1/15/2019 Reviewed [file giz034_reviewer-2-report-revision-1.pdf]

Reviewer Report

Title: Fast and accurate relatedness estimation from high throughput sequencing data in the presence of inbreeding

Version: Revision 1 **Date:** 1/15/2019

Reviewer name: Jan Graffelman

Reviewer Comments to Author:

The authors have updated their manuscript, taking most of the suggestions of the reviewers satisfactorily into account. I have some additional comments, mostly minor, that may be of help for the authors to further improve their manuscript.
Abstract: "In the case low coverage" --> "in the case of low coverage"
Pg. 2, l. 4. The authors may wish to add "respectively, and known as Cotterman's coefficients (Cotterman, 1941)."
Pg. 2, l. 28. "as well as inbreeding coefficient" --> "as well as inference of the inbreeding coefficients"
Pg. 2, l. 6, 2nd col. "population frequencies" --> "population allele frequencies"
Pg. 2, l. 30, 2nd col. "Bernoulli trials" --> "independent Bernoulli trails"
Pg. 2, l. 30, 2nd col. "given site." The authors may wish to add: "given site, implying the data is generated under the assumption of Hardy-Weinberg equilibrium."
Pg. 3, l. 8: "estimated from" --> "estimated by"
Pg. 3, l. 18: J7; the "7" should be a subscript.
Pg. 3, l. 33: It is not clear what is meant with "confident estimates". Improved estimates?
Pg. 3, l. 60: Supplementary figure 4. This is an important application showing that the proposed estimation procedure seems to work well in practice. I suggest the figure not to be supplementary, but part of the main manuscript. The documented relationships of the six panels should be clearly indicated, for instance by putting (PO), (FS) or whatever corresponds in the banner of each panel behind the identifiers of each pair. Documented relationships could be mentioned in the text or the caption of the figure as well.
The authors have apparently been a bit careless in their update of the bibliography. Please address the following issues:
16: the author name is misspelled.
17. This book is not from 2015, but from 1974. Please check out the correct reference.
19. The author, the 1000G Consortium, is not correctly spelled out.
25. Surnames not fully in uppercase.
References:
Cotterman, C. W. (1941). Relative and human genetic analysis. The Scientific Monthly, 53, 227-234.

Level of Interest

Please indicate how interesting you found the manuscript: Choose an item.

Quality of Written English

Please indicate the quality of language in the manuscript: Choose an item.

Declaration of Competing Interests

Please complete a declaration of competing interests, considering the following questions:

- Have you in the past five years received reimbursements, fees, funding, or salary from an organisation that may in any way gain or lose financially from the publication of this manuscript, either now or in the future?
- Do you hold any stocks or shares in an organisation that may in any way gain or lose financially from the publication of this manuscript, either now or in the future?
- Do you hold or are you currently applying for any patents relating to the content of the manuscript?
- Have you received reimbursements, fees, funding, or salary from an organization that holds or has applied for patents relating to the content of the manuscript?
- Do you have any other financial competing interests?
- Do you have any non-financial competing interests in relation to this paper?

If you can answer no to all of the above, write 'I declare that I have no competing interests' below. If your reply is yes to any, please give details below.

I declare that I have no competing interests

I agree to the open peer review policy of the journal. I understand that my name will be included on my report to the authors and, if the manuscript is accepted for publication, my named report including any attachments I upload will be posted on the website along with the authors' responses. I agree for my report to be made available under an Open Access Creative Commons CC-BY license (http://creativecommons.org/licenses/by/4.0/). I understand that any comments which I do not wish to be included in my named report can be included as confidential comments to the editors, which will not be published.

I agree to the open peer review policy of the journal

To further support our reviewers, we have joined with Publons, where you can gain additional credit to further highlight your hard work (see: https://publons.com/journal/530/gigascience). On publication of this paper, your review will be automatically added to Publons, you can then choose whether or not to claim your Publons credit. I understand this statement.

Yes Choose an item.
